# Unravelling the Gut Microbiome Role in Cardiovascular Disease: A Systematic Review and a Meta-Analysis

**DOI:** 10.3390/biom14060731

**Published:** 2024-06-20

**Authors:** Diana Martins, Cláudia Silva, António Carlos Ferreira, Sara Dourado, Ana Albuquerque, Francisca Saraiva, Ana Beatriz Batista, Pedro Castro, Adelino Leite-Moreira, António S. Barros, Isabel M. Miranda

**Affiliations:** 1 Cardiovascular R&D Centre—UnIC@RISE, Department of Surgery and Physiology, Faculty of Medicine, University of Porto, 4200-319 Porto, Portugal; 2 Department of Neurology, São João Hospital Center, Faculty of Medicine, University of Porto, 4200-319 Porto, Portugal

**Keywords:** microbiota, microbiome, dysbiosis, metabolites, cardiovascular disease, trimethylamine N-oxide, systematic review, meta-analysis

## Abstract

A notable shift in understanding the human microbiome’s influence on cardiovascular disease (CVD) is underway, although the causal association remains elusive. A systematic review and meta-analysis were conducted to synthesise current knowledge on microbial taxonomy and metabolite variations between healthy controls (HCs) and those with CVD. An extensive search encompassing three databases identified 67 relevant studies (2012–2023) covering CVD pathologies from 4707 reports. Metagenomic and metabolomic data, both qualitative and quantitative, were obtained. Analysis revealed substantial variability in microbial alpha and beta diversities. Moreover, specific changes in bacterial populations were shown, including increased *Streptococcus* and *Proteobacteria* and decreased *Faecalibacterium* in patients with CVD compared with HC. Additionally, elevated trimethylamine N-oxide levels were reported in CVD cases. Biochemical parameter analysis indicated increased fasting glucose and triglycerides and decreased total cholesterol and low- and high-density lipoprotein cholesterol levels in diseased individuals. This study revealed a significant relationship between certain bacterial species and CVD. Additionally, it has become clear that there are substantial inconsistencies in the methodologies employed and the reporting standards adhered to in various studies. Undoubtedly, standardising research methodologies and developing extensive guidelines for microbiome studies are crucial for advancing the field.

## 1. Introduction

The human gut harbours a diverse collection of microorganisms, including bacteria, archaea, viruses, and fungi, collectively called microbiota. Together with their genomes, microbial structures, metabolites, and environment, they form the gut microbiome. The prevalence of bacteria, particularly those belonging to the *Firmicutes* and *Bacteroidetes* phyla, is noteworthy in the context of the microbiome. The ratio of these two phyla (F/B) has been identified as a useful indicator of the microbiome’s status and has been associated with a range of diseases [1]. The classification system for microbiome enterotypes is based on the composition of microorganisms and has been shown to offer clinical advantages. Moreover, analysing the structure of microbiota through measurements of alpha diversity, which expresses the microbial richness in a single sample, and beta diversity, which measures differences in the microbial composition between samples, offers valuable insights [2].

The gut microbiome plays a fundamental role in digestion, epithelial barrier formation, protection against pathogens, and immune system regulation. These functions involve direct interactions with host cells or key microbe-produced metabolites, suggesting that the gut microbiome acts as an endocrine organ [3]. Metabolomic research has uncovered and examined several gut microbiota-derived metabolites. These metabolites can be classified based on their source and synthesis mode to comprehensively understand their characteristics and functions. The gut microbiota plays a crucial role in the production of various metabolites, including short-chain fatty acids (SCFAs), which are formed as a result of the fermentation of undigested carbohydrates. Additionally, trimethylamine (TMA), which is derived from food components such as carnitine and choline, is enzymatically converted to trimethylamine-N-oxide (TMAO) in the liver. Furthermore, host metabolites, such as bile acids (BAs), undergo modification by gut bacteria. Lastly, gut bacteria also synthesise *de novo* metabolites, including branched-chain amino acids, vitamins, and polyamines [4].

The emergence of dysbiosis, a state of microbial community imbalance, is primarily attributed to factors such as a poor diet, elevated stress levels, inadequate physical activity, and the use of antibiotics. This imbalance arises when microorganisms cannot maintain the equilibrium necessary for optimal functioning [5]. Over the past few decades, studies have indicated the potential involvement of dysbiosis in various illnesses and disorders, including cardiovascular disease (CVD) [6]. CVD, which encompasses a range of conditions affecting the cardiovascular system, continues to be a major contributor to global mortality and disability rates despite sustained efforts to mitigate its impact. Hypertension (HTN), diabetes, smoking, genetics, and lifestyle are risk factors, and atherosclerosis (AS) is a major underlying cause of certain CVD [7]. Although the causal association between the gut microbiome and CVD remains unknown, meaningful progress has been made, mainly through animal studies. The gut microbiome contributes to CVD pathophysiology, particularly atherogenesis, by modulating inflammatory and metabolic pathways. Dysbiosis-induced intestinal permeability increases lipopolysaccharide (LPS) translocation, triggering inflammation through the induction of pro-inflammatory cytokines [8]. In addition, LPS and TMAO contribute to oxidative stress, leading to endothelial dysfunction. At the same time, TMAO and BAs impact blood lipid composition, contributing to AS progression and elevating the risk of adverse cardiovascular events [9].

Modulation of the gut microbiome through dietary modifications and pre-and probiotic supplementation has the potential to influence CVD [10]. These interventions can manipulate the microbial composition and activity, decrease harmful metabolites, improve lipid profiles, and reduce inflammation. Despite their potential benefits, addressing challenges such as the complexity and inter-individual variability of the microbiome, as well as a limited understanding of microbial functions, is essential [11]. Moreover, drugs can also influence the composition and function of gut microbes, including oral cardiac drugs. However, the interaction between the gut microbiome and drugs is bidirectional, as bacteria can metabolise medications, altering their mechanisms of action and affecting side effects [12].

This review comprehensively synthesises scientific insights into the implications of the gut microbiome for CVD. By conducting a systematic review and meta-analysis of 67 published studies, including cross-sectional and case-control studies with a total sample size of 4855 controls and 6090 patients, we aimed to reveal differences in microbial taxonomy and metabolite levels between patients with CVD and healthy individuals. This study contributes to the understanding of the functional role of the gut microbiome in CVD pathophysiology, thereby facilitating the development of innovative therapeutic strategies and advancements in prevention and early detection techniques.

## 2. Materials and Methods

### 2.1. Search Strategy and Study Selection

This meta-analysis was registered on the Open Science Framework (OSF) [13]. Additionally, this study followed the Preferred Reporting Items for Systematic Reviews and Meta-Analyses (PRISMA) guidelines [14]. The compliance checklists for PRISMA can be found in Tables S1 and S2. Our primary aim was to investigate the relationship between the gut microbiome and various CVDs compared with healthy controls (HCs).

The literature search was conducted using three electronic databases, PubMed, Scopus, and Web of Science, from inception until 20 March 2023. To extract all relevant articles from the databases, the search strategy was based on the following query: “cardiovascular disease” AND “microbiota” AND “metabolites” combined with Medical Subject Headings (MeSH). Moreover, our search strategy incorporated a filter for the “humans” keyword to ensure the inclusion of studies addressing human subjects. Exclusion filters were applied to restrict the results to research articles by excluding certain types of publications, namely “comment”, “editorial”, “letter”, “meta-analysis”, “review,” and “systematic review.” Table S1 provides a detailed overview of the search process.

The reviewers used Rayyan’s free web tool [15] to screen the retrieved records. After eliminating duplicates, three reviewers (A.A., D.M. and S.D.) independently screened the titles and abstracts of the papers. Five reviewers, A.A., A.F., C.S., D.M. and S.D., screened the full-text records for eligible studies. They included studies that referred to metagenomic and metabolomic analysis. Any discrepancies were resolved through discussions among the reviewers.

### 2.2. Data Extraction

Information regarding the study characteristics was extracted using a self-developed data extraction table. The table records the authors’ names, publication year, country, diagnostic type, sample size, cohort variables (e.g., age, sex, and body mass index [BMI]), and a brief description of the included patients with CVD and HC (Table S2). We also compiled data on laboratory parameters from healthy and diseased subjects from studies that provided this information (Table S3).

For studies that included metagenomic analysis, we retrieved information regarding the DNA extraction kit employed, the applied microbial profiling method, the corresponding analysed sequencing region, and the approach used for sequence clustering (Table S4). Regarding the studies that included metabolomic analysis, we registered information about the sample type, analytical technique used, and identified metabolite types (Table S5).

The review involved qualitative and quantitative synthesis of metagenomic data, including alpha and beta diversities; the relative abundance of each bacterium; and metabolomic data, including metabolite measurements. Only the reported statistically significant differences were employed for analysis. Additionally, we extracted values in the form of mean/standard deviation and median/interquartile range for quantitative synthesis. These values were obtained from the articles, Supplementary Materials, and the freely available WebPlotDigitizer V4 tool [16], which facilitated data extraction from images. We contacted the corresponding author to request data sharing if data were unavailable. The median/interquartile range values were estimated as mean/standard deviation to standardise the data for further analysis [17]. Five researchers (A.F., B.B., C.S., D.M. and S.D.) independently extracted data with subsequent comparisons to guarantee accuracy.

### 2.3. Data Analysis

In this meta-analysis, we used data from at least three studies. Bacteria and metabolites that lacked sufficient quantitative information for the analysis were excluded. Meta-analyses and their respective forest plots were elaborated using Review Manager Web software [18], using the random-effects model with inverse variance to calculate the standardised mean differences (SMD) and 95% confidence intervals. A random-effects model was also employed to estimate the pooled effect size owing to the expected heterogeneity among the studies. Heterogeneity was assessed using the Tau^2^, Chi^2^, and I^2^ statistics, with statistical significance at *p* < 0.05. The Z-test estimated the overall effect size.

### 2.4. Quality Assessment

The internal validity and potential bias of the included studies were assessed using the National Health, Lung, and Blood Institute (NHLBI) study quality assessment tool for Observational Cohort and Cross-Sectional Studies and Case-Control Studies, as shown in Tables S6 and S7, respectively [19]. Four reviewers (A.F., C.S., D.M. and S.D.) independently assessed the studies’ quality and addressed discrepancies through discussion.

## 3. Results

### 3.1. Study Selection and Characteristics

**Figure 1:**
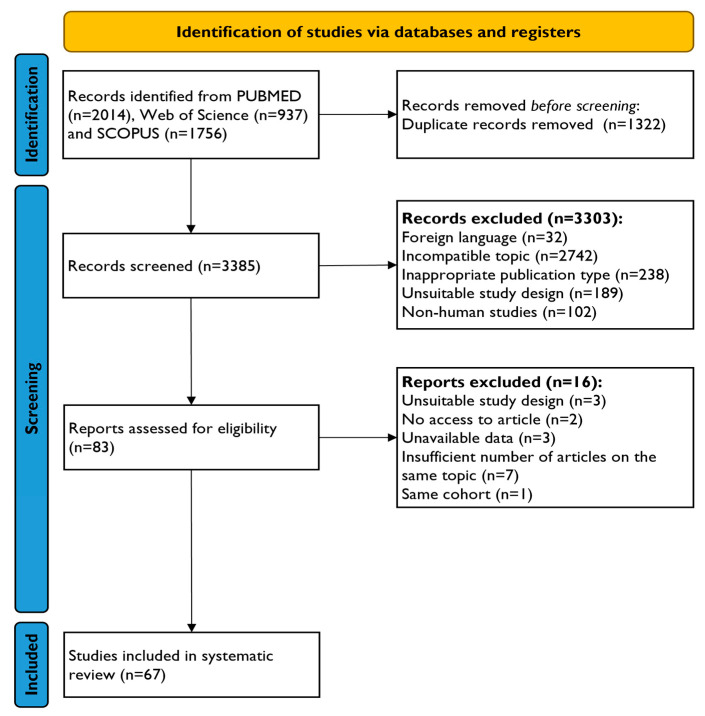
Study selection process for systematic review and meta-analysis. This PRISMA flow chart outlines each process stage, depicting the number of records involved and clarifying the inclusion and exclusion criteria.

A total of 4707 articles were initially identified using a prespecified query. After removing 1322 duplicates, 3385 records underwent title and abstract screening. Exclusions included 32 non-English articles, 2742 unrelated to this work’s purposes, 238 non-original studies, 189 with different designs, and 102 without human subjects. The remaining 83 studies were further assessed, leading to exclusion for reasons such as inaccessibility, non-pre-specified study design, unavailable data, and lack of comparable data. Ultimately, 67 reports [20,21,22,23,24,25,26,27,28,29,30,31,32,33,34,35,36,37,38,39,40,41,42,43,44,45,46,47,48,49,50,51,52,53,54,55,56,57,58,59,60,61,62,63,64,65,66,67,68,69,70,71,72,73,74,75,76,77,78,79,80,81,82,83,84,85,86] met the eligibility criteria and were included in this study. The study selection process is illustrated in Figure 1.

Of the 67 eligible studies from 2012 to 2023, the analysis encompassed cross-sectional (n = 17) and case-control (n = 50) studies. Quality assessment revealed studies rated as “Good” (n = 27), “Fair” (n = 39), and “Poor” (n = 1). The majority of the studies centred on Asia (n = 55), mainly focusing on China (n = 47), while the remaining studies were conducted in Europe (n = 10), Australia (n = 6), and the United States (n = 1). Categorised by CVD type, the studies included acute coronary syndrome (ACS) (n = 9), AS (n = 4), atrial fibrillation (AF) (n = 5), coronary artery disease (CAD) (n = 16), heart failure (HF) (n = 9), HTN (n = 6), and stroke (n = 18). The analysis included 6090 patients with a mean of 62.3 (±12.6) years, 67% males, and a mean BMI of 25.5 (±3.7). The HC group included 4855 individuals with a mean age of 59.6 (±10.4), 57% males, and a mean BMI of 24.6 (±3.1).

Patients were included based on their disease history and clinical examinations, such as electrocardiography, coronary angiography, computed tomography, and magnetic resonance imaging. Specific inclusion criteria were applied for certain cardiovascular conditions, such as cardiac troponin levels for ACS and blood pressure thresholds for HTN. Some studies required the participants to provide written informed consent. The control group comprised individuals without specific medical conditions, with negative cardiac examination results, normal biomarker levels, and age- and sex-matched individuals, some of whom were enrolled voluntarily. The exclusion criteria included prior gastrointestinal surgery, active infections, inflammatory bowel disease, autoimmune diseases, malignancy, history of stroke, renal failure, hepatic disorders, digestive diseases, smoking, alcohol use, and antibiotic or probiotic intake within a specified timeframe before sample collection. Table S2 provides a comprehensive overview of the characteristics of the included studies.

### 3.2. Methods of Metagenomic and Metabolomic Analysis

Among the 67 eligible studies, 24 conducted comprehensive microbiota analyses, nine focused on metabolic analysis, and 34 performed both microbiota and metabolite analyses. Microbiota-focused studies used DNA extraction kits primarily from QIAGEN (Hilden, NRW, Germany) (n = 20) and TIANGEN (Beijing, China) (n = 9). Techniques included 16S rRNA gene sequencing (n = 40) and shotgun metagenomic sequencing (n = 16), with the V3–V4 hypervariable region of the bacterial 16S rRNA gene being the most analysed (n = 29). SILVA (n = 19) was the predominant mapping database used. In contrast, 43 studies concentrated on microbial-derived metabolite measurements using various chromatographic techniques coupled with mass spectrometry, with liquid chromatography-mass spectrometry (LC-MS) being the most employed (n = 32). Blood samples (serum and plasma) were predominant (n = 27), and TMAO emerged as a widely investigated metabolite, as assessed in multiple studies (n = 22). Tables S4 and S5 provide the detailed characteristics of the studies performing microbiota and metabolic analyses, respectively.

### 3.3. Qualitative Synthesis

**Figure 2:**
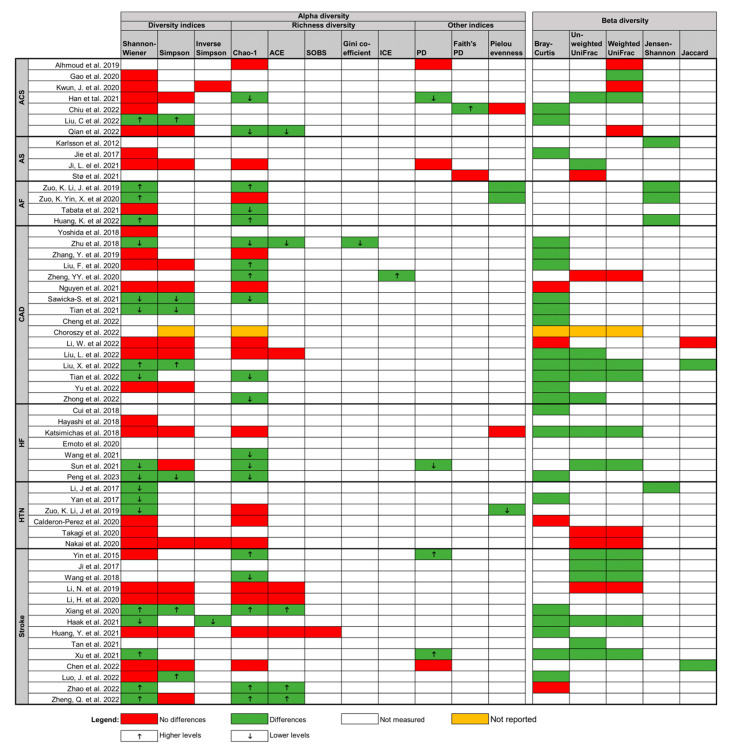
Qualitative analysis of microbial diversity data across different cardiovascular disease (CVD) types [20,22,23,24,25,26,27,28,31,32,33,34,35,36,37,38,39,40,41,42,43,44,45,46,47,48,49,50,51,52,53,55,56,57,59,60,62,63,64,66,67,69,70,71,72,73,74,75,76,78,79,80,81,82,83,84,85,86]. Green indicates significant differences in diversity between CVD patients and healthy controls (HC), red signifies no significant differences, yellow represents unreported results, and white denotes unmeasured metrics. The upward arrow (↑) indicates increased diversity, and the downward arrow (↓) indicates decreased diversity. Abbreviations: ACE = Abundance-based Coverage Estimator, ACS = acute coronary syndrome, AF = atrial fibrillation, AS = atherosclerosis, CAD = coronary artery disease, HF = heart failure, HTN = hypertension, ICE = Incidence-based Coverage Estimator, PD = Phylogenetic Diversity, SOBS = number of observed species.

Significant variability was noted among 58 microbiota analysis studies; however, most reported a statistically significant relationship between CVD and gut microbiota composition (Figure 2). Shannon–Wiener diversity (n = 45) and Simpson diversity (n = 24) were the primary metrics for alpha-diversity assessment, often in conjunction with Chao-1 richness (n = 35). Of 53 studies comparing gut microbiota between HC and CVD patients, eight reported higher alpha diversity in CVD patients, 11 indicated lower alpha diversity, 22 found no statistically significant differences, 11 showed contradictory results between tests, and one did not report results. Beta diversity, mainly assessed by Bray–Curtis dissimilarity (n = 28) and UniFrac distances (n = 39), was reported in 50 studies. Among these, 37 found significant differences in the gut microbiota between patients with CVD and HC, while 12 did not show significant differences, and one did not report results.

**Figure 3:**
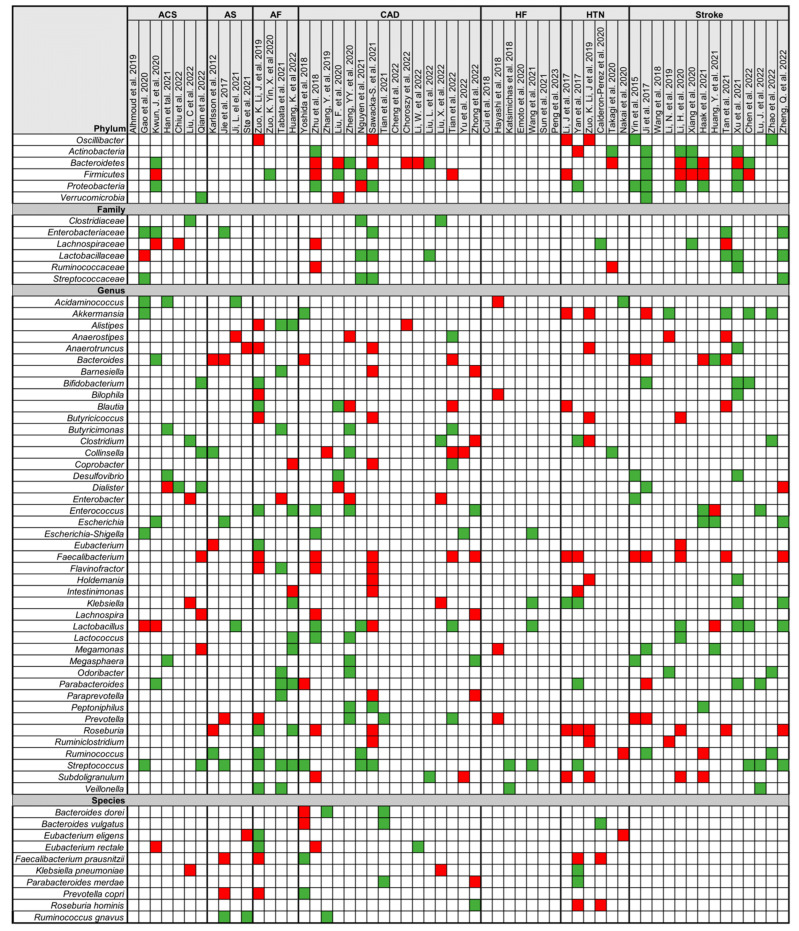
Qualitative analysis of bacterial relative abundance data. Qualitative analysis of bacterial relative abundance data [20,22,23,24,25,26,27,28,31,32,33,34,35,36,37,38,39,40,41,42,43,44,45,46,47,48,49,50,51,52,53,55,56,57,59,60,62,63,64,66,67,69,70,71,72,73,74,75,76,78,79,80,81,82,83,84,85,86]. Rows represent bacteria taxa from phylum to species, while columns correspond to studies concerning different types of cardiovascular disease (CVD). Green indicates significantly higher relative abundance in CVD patients, red denotes significantly lower relative abundance, and white flags unquantified taxa. Abbreviations: ACS = acute coronary syndrome, AF = atrial fibrillation, AS = atherosclerosis, CAD = coronary artery disease, HF = heart failure, HTN = hypertension.

In 58 studies, significant differences in the relative abundance between patients with CVD and HC were observed (Figure 3). Notably, *Actinobacteria* demonstrated an increased relative abundance in CVD patients compared to HC in six out of seven studies where it was analysed (6/7). Similarly, *Proteobacteria* (9/10) showed increased relative abundances in the CVD group at the phylum level. However, findings regarding the *Firmicutes* and *Bacteroidetes* phyla were inconsistent. Due to study heterogeneity, the ratio between these phyla (F/B), which serves as a marker for microbial imbalance, is of uncertain reliability. At the genus level, all studies involving patients with CVD have identified a consistent decrease in *Faecalibacterium* (13/13). Enrichment of *Lactococcus* (4/4) and *Streptococcus* (15/15) was noted, indicating a significant association with CVD. The abundance of the *Lactobacillaceae* family (6/7) and its *Lactobacillus* genus (9/13), along with the *Clostridiaceae* family (3/3) and its *Clostridium* genus (4/6), increased in patients with CVD. As well as the rise of the *Enterobacteriaceae* family in the CVD group (6/6), its genera *Klebsiella* (6/8) and *Escherichia* (5/5) also displayed positive associations, while the genus *Enterobacter* (4/5) showed negative association. Genera such as *Bifidobacterium* (5/5), *Butyricimonas* (4/4), *Desulfovibrio* (4/4), *Enterococcus* (7/8), *Megasphaera* (4/4), *Parabacteroides* (6/8), *Ruminococcus* (5/7), and *Veillonella* (4/4) were enriched in patients with CVD, whereas *Bacteroides* (8/10), *Blautia* (4/6), *Butyricicoccus* (4/4), *Oscillibacter* (4/6), *Roseburia* (9/11), and *Subdoligranulum* (6/7) were reduced in diseased individuals. Variations were observed among different CVD pathologies, with distinct increases in the genera *Roseburia*, *Prevotella*, and *Oscillibacter* observed exclusively in AF, CAD, and stroke.

**Figure 4:**
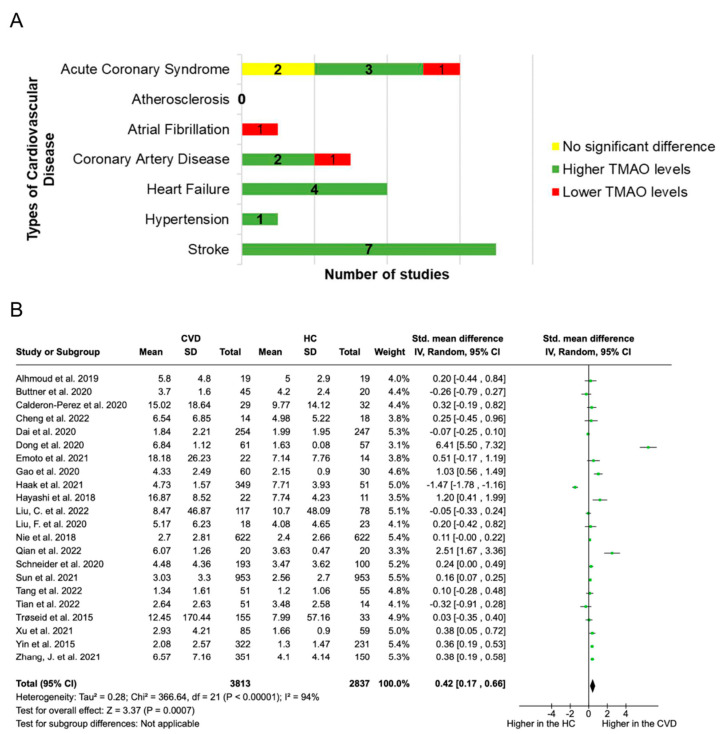
Trimethylamine N-Oxide (TMAO) levels vary across different types of cardiovascular disease (CVD). (**A**) Qualitative analysis. Green bars indicate studies with a significant increase in TMAO levels in patients with CVD compared to healthy controls (HC), red bars signify a significant decrease, and yellow bars represent studies reporting no significant differences. (**B**) Quantitative analysis. For each group (CVD and HC), the mean relative abundance (Mean), standard deviation (SD), and sample size (Total) are provided [20,21,24,30,31,32,35,49,54,56,58,60,65,72,74,77]. The green square markers denote TMAO levels in studies comparing individuals with and without CVD. The horizontal black lines represent the 95% confidence intervals of the study result. The diamond-shaped data marker reflects the pooled estimate (standard mean difference = 0.42), emphasising higher TMAO levels in individuals with CVD than those without.

Of the 67 included studies, 43 conducted metabolomic analyses, with only 22 analysing microbial-derived metabolites in individuals with CVD and HC. This analysis focused on TMAO levels, considering three or more studies for each pathology. Among these 22 studies, 16 reported significantly increased TMAO levels in patients with CVD [ACS (n = 3), CAD (n = 2), HF (n = 4), HTN (n = 1), and stroke (n = 7)]. Three studies showed significantly lower TMAO levels [ACS (n = 1), AF (n = 1), and CAD (n = 1)]. Two studies found no significant differences [ACS (n = 2)]. Overall, patients with CVD tended to have higher TMAO levels compared to HC. When analysing different pathologies separately, only ACS, HF, and stroke had a sufficient number of studies (≥3), with elevated TMAO concentrations notably detected in patients with HF (4/4) and stroke (7/7) (Figure 4A). Such analyses were impossible for HTN (1 study) and AS (zero studies) due to insufficient studies.

### 3.4. Quantitative Synthesis

**Figure 5:**
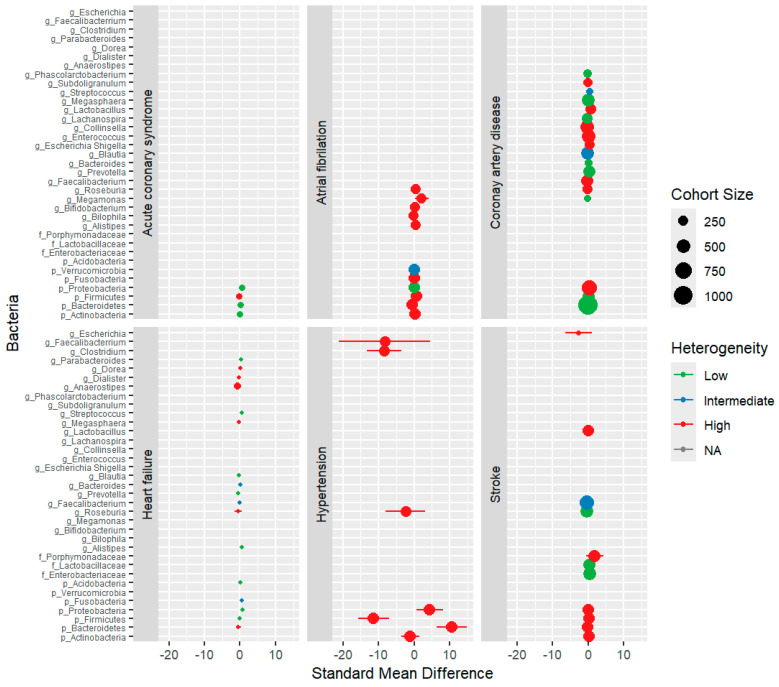
Relative abundance of bacteria across different types of cardiovascular disease (CVD). Forest plot analysis of standard mean differences (SMD) in bacterial relative abundance between patients with CVD and healthy controls (HC). The size of each circle corresponds to the cohort size, reflecting study participant numbers. Horizontal lines represent 95% confidence intervals for individual study results. The heterogeneity level is indicated by colour-coding: I^2^ values of less than 25% (low, green), 25–50% (intermediate, blue), and over 50% (high, red).

**Figure 6:**
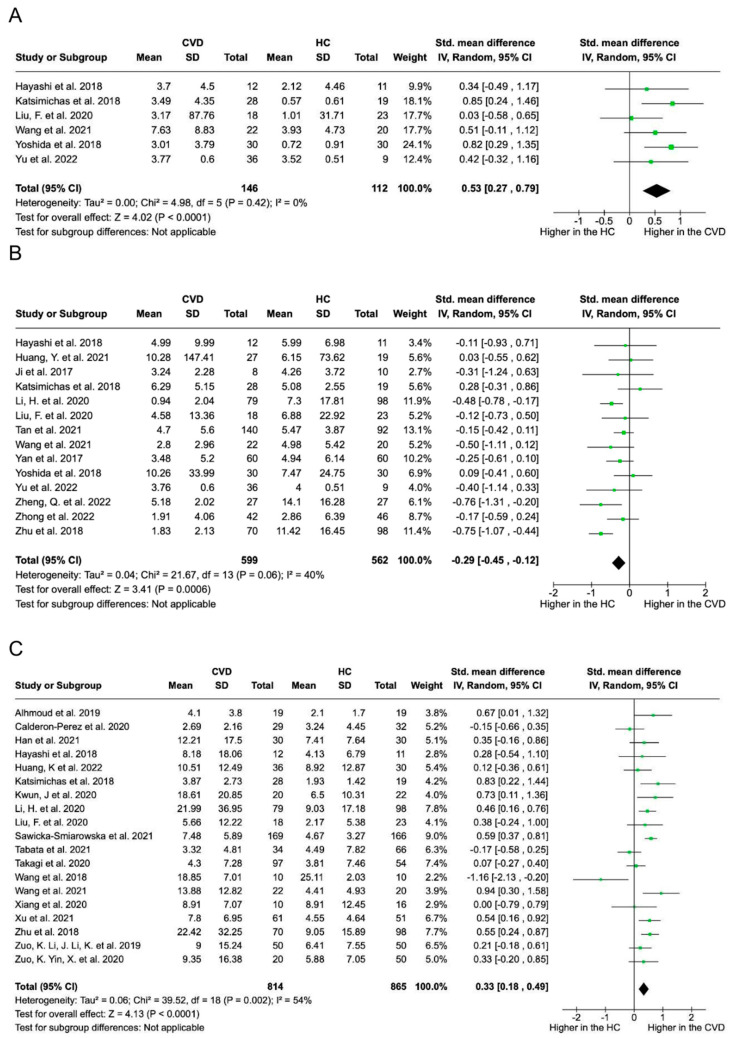
Forest plots of bacteria genus relative abundance. Quantitative analysis compared the abundance of bacterial genera in individuals with and without cardiovascular disease (CVD). For each group, healthy control (HC) and CVD, the mean relative abundance (Mean), standard deviation (SD), and sample size (Total) are provided. The green square markers represent the relative abundance of bacteria, with horizontal black lines indicating the 95% confidence intervals of the study results. The diamond-shaped data markers show higher *Streptococcus* (**A**) and *Proteobacteria* (**C**) levels (SMD = 0.53 and 0.33, respectively) in the CVD group. Conversely, *Faecalibacterium* (**B**) decreased (SMD = −0.29) in the CVD group compared with HC [20,22,34,35,36,37,39,42,43,49,57,62,63,64,69,70,71,73,76,80,82,83,84,86,87,88,89].

Quantitative analyses were conducted by considering taxonomic levels only when a minimum of three independent relative abundances were available. Figure 5 presents the compiled results of individual meta-analyses for each bacterium across different CVD types. At the phylum level, *Proteobacteria* exhibited a significantly higher prevalence in ACS (*p* = 0.002, standardised mean difference [SMD] = 0.55) and HF (*p* = 0.0002, SMD = 0.75, no heterogeneity). In contrast, CAD showed a higher prevalence of *Proteobacteria* (*p* = 0.03, SMD = 0.37, 70% heterogeneity). *Fusobacteria* increased in the HF group (*p* = 0.02, SMD = 0.56, 33% heterogeneity), while *Bacteroidetes* decreased in the AF group (*p* = 0.006, SMD = −0.56, 67% heterogeneity). At the family level, stroke patients exhibited an increase in *Enterobacteriaceae* (*p* < 0.00001, SMD = 0.49, no heterogeneity) and *Lactobacillaceae* (*p* = 0.006, SMD = 0.29, no heterogeneity). At the genus level, HF displayed an increase in *Streptococcus* (*p* = 0.002, SMD = 0.60, no heterogeneity) and *Alistipes* (*p* = 0.01, SMD = 0.48, no heterogeneity). AF showed an increase in *Megamonas* (*p* = 0.03, SMD = 2.18, 97% heterogeneity), whereas CAD showed an increase in *Lactobacillus* (*p* = 0.04, SMD = 0.62, 79% heterogeneity). Conversely, *Prevotella* decreased in HF (*p* = 0.04, SMD = −0.40, no heterogeneity), *Lachnospira* decreased in CAD (*p* = 0.003, SMD = −0.35, no heterogeneity), and *Roseburia* decreased in stroke (*p* = 0.0002, SMD = −0.35, no heterogeneity). Likewise, *Faecalibacterium* was decreased in stroke patients (*p* = 0.009, SMD = −0.33, 37% heterogeneity). Across all pathologies, patients with CVD demonstrated elevated levels of *Streptococcus* (*p* < 0.0001, SMD = 0.53, no heterogeneity) and *Proteobacteria* (*p* = 0.0008, SMD = 0.69, 93% heterogeneity), along with a reduction in *Faecalibacterium* (*p* = 0.0006, SMD = −0.29, 40% heterogeneity). Note that the latter two associations excluded an outlier study from the analysis. Figure 6 provides a visual representation of the findings.

Furthermore, TMAO was the only microbial-derived metabolite subjected to quantitative analysis, as no other metabolite had reported values in at least three studies. The meta-analysis (Figure 4B), which included 22 studies comparing CVD and HC individuals, revealed increased TMAO levels in the CVD group (*p* = 0.0007; SMD = 0.42, 94% heterogeneity).

In our analysis of 67 studies, 47 provided biochemical data characterising the study cohorts. Meta-analyses conducted on parameters with at least three reported results revealed significant differences (Figure S1). Elevated fasting glucose (FG) levels were observed in patients with ACS (*p* = 0.001, SMD = 1.01, 88% heterogeneity) and CAD (*p* = 0.02, SMD = 0.42, 82% heterogeneity), as well as those with HTN (*p* = 0.02, SMD = 0.25, no hete-rogeneity). Individuals with CAD (*p* = 0.01, SMD = 0.67, 93% heterogeneity) and HTN (*p* = 0.004; SMD = 0.34; no heterogeneity) consistently exhibited elevated triglyceride (TG) levels. Conversely, decreased total cholesterol (TC) levels were associated with certain cardiovascular conditions, including AF (*p* < 0.00001, SMD = −0.83, no heterogeneity), HF (*p* = 0.05, SMD = −0.93, 90% heterogeneity), and AS (*p* < 0.00001, SMD = −1.10, no heterogeneity). Additionally, patients with AS exhibited a significant decrease in low-density lipoprotein cholesterol (LDL-C) levels (*p* = 0.0001, SMD = −1.21, 68% heterogeneity), while high-density lipoprotein cholesterol (HDL-C) levels were reduced in patients with stroke (*p* < 0.0001, SMD = −1.20, 98% heterogeneity) and CAD (*p* < 0.0001, SMD = −0.54, 73% heterogeneity). Furthermore, creatinine levels were increased in patients with ACS (*p* = 0.0009, SMD = 0.32, no heterogeneity), with an excluded outlier to address the observed heterogeneity.

## 4. Discussion

This study analysed human studies that evaluated gut microbiome alterations in patients with CVD. As the prevalence of CVD continues to increase, posing a significant burden on public health systems worldwide, understanding its underlying mechanisms is crucial. Several studies have revealed a consistent connection between the human gut microbiome and CVD pathophysiology, with microorganisms playing a role in metabolising dietary components and generating metabolites that exacerbate or mitigate CVD risk factors. Hence, exploring the intricate roles of microbiota and metabolites provides a promising avenue for devising novel preventive and therapeutic strategies to reduce the escalating CVD epidemic and enhance public health.

This meta-analysis and systematic review of 67 studies explored the differences in faecal microbiota and metabolites between HCs and patients with CVD. These comprehensive findings offer the latest insights into the connection between cardiovascular health and the gut microbiome. Despite the variations in the study parameters, we present a comprehensive synthesis of microbial taxonomy and metabolite measurements.

Our analysis included various alpha and beta diversity metrics to compare the gut microbiota datasets between the CVD and HC groups. Notably, variations in metric choices impacted the results. We observed significant variability in microbial diversity, with over 40% of studies reporting no significant differences in alpha diversity between CVD patients and HC. However, beta diversity analysis revealed significant distinctions in over 70% of studies, posing challenges for drawing definitive conclusions. Additionally, the application of varying significance thresholds impeded direct comparisons between studies. Nonetheless, the overall analysis of microbial diversity revealed differences in the gut microbiota composition between HC and individuals with CVD.

Elevated levels of the phylum *Proteobacteria*, linked to dysbiosis and disease state, are notably present in individuals with cardiac events [89]. Moreover, *Proteobacteria* translocation from the gut to the bloodstream, with its outer membrane composed of LPS, leads to endotoxemia, immune system activation, and inflammation, which are crucial factors in cardiovascular conditions [8]. Furthermore, overabundance of *Proteobacteria* is associated with epithelial dysfunction, a CVD condition [9]. These bacteria influence cardiovascular health through genes involved in TMAO formation, thereby affecting cholesterol metabolism, blood pressure regulation, and vascular function [20].

Despite these variations, this review across different CVDs consistently found higher levels of *Streptococcus* and *Streptococcaceae*, along with a decrease in *Faecalibacterium* and its species *Faecalibacterium prausnitzii*, in patients with CVD than in HCs. This consistent pattern suggests that a significant microbial shift is associated with CVD.

*Streptococcus*, typically beneficial, can become harmful when the immune system weakens, triggering inflammation and potentially exacerbating CVDs. Studies have indicated associations between elevated *Streptococcus* levels and the development, progression, and poor prognosis of CVDs [90]. Its association with AS, a prominent contributor to CVD, is well established. For instance, *Streptococcus* species found in both the gut and atherosclerotic plaques suggest their involvement in AS development [91]. Moreover, Atarashi et al. (2017) observed increased gut colonisation by *Streptococcus* in patients with CAD [92]. This opportunistic pathogen is linked to HTN severity and produces neurotransmitters that affect vascular tone, thereby contributing to HTN [93]. In patients with AF, *Streptococcus* overgrowth, along with reduced *Faecalibacterium*, may contribute to microbial imbalance [94]. Nevertheless, *Streptococcus* is associated with SCFAs and TMA synthesis, which are crucial in cardiovascular physiological processes [95]. Additionally, *Streptococcus* showed a positive correlation with FG and a negative correlation with TC, consistent with the observed high FG and low TC levels in patients with CVD in our study [96].

The reduced abundance of *Faecalibacterium* in patients with CAD suggests a lower concentration of SCFAs, which hinders the energy supply for intestinal cells and compromises the gut barrier by impairing Toll-like receptor 2 in tight junction formation [83]. Li et al. (2021) observed that secondary BAs promote the development of this genus, suggesting their involvement in bile acid metabolism [97]. Although our findings do not align with the existing literature owing to limited metabolite data, a consistent trend was observed for TMAO. Several studies have highlighted a negative correlation between *F. prausnitzii* and TMAO production [28]. Our analysis supports this finding, revealing decreased *Faecalibacterium* levels and elevated TMAO levels in patients with CVD.

Research on *F. prausnitzii*-treated mice indicates cardiovascular benefits such as enhanced cardiac function, lower plasma lipid levels, reduced atherosclerotic plaque formation, and relief from post-stroke neurological deficits and inflammation [88].

Likewise, human studies associate higher *Faecalibacterium* levels with lower arterial stiffness [98]. Additionally, *Faecalibacterium* negatively correlates with CVD risk factors, including inflammatory markers such as high-sensitivity C-reactive protein and interleukin-6, and positively correlates with tumour necrosis factor-α [99]. Concerning lipid-related markers, *Faecalibacterium* was negatively associated with LDL-C, TC, and TG and positively associated with HDL-C [100]. However, our findings differ from those in the literature, showing a decrease in HDL-C levels and an increase in TG levels. *F. prausnitzii* also negatively correlates with insulin resistance markers, but our results indicate elevated FG levels in patients with CVD [101]. Insulin resistance, reflected in increased FG, is a hallmark of pre-diabetes and type 2 diabetes, both of which are risk factors for CVD. This bacterium is also negatively correlated with uric acid levels [24], and our findings revealed elevated creatinine levels, suggesting kidney dysfunction, which is closely linked to CVD risk.

The gut microbiota impacts the overall metabolic balance and regulates serum lipid and glucose levels [102]. In this meta-analysis examining various biochemical parameters related to CVD, patients showed increased levels of FG and TG compared to HCs. Conversely, TC LDL-C and HDL-C levels were reduced. Notably, the acid-lactic-producing bacterium *Streptococcus* exhibits a strong positive correlation with CVD risk factors, including blood glucose, HDL-C, and ApoAI (a significant HDL component); however, it is negatively correlated with TC [96]. No direct associations were found between this opportunistic bacterium and LDL-C and TG [103].

The well-established link between gut microbiota and CVD involves the TMAO metabolite pathway, although the exact mechanism is not fully understood. Our meta-analysis and systematic review confirmed elevated plasma TMAO levels in patients with CVD compared to HC, particularly in individuals with ACS, CAD, HF, and stroke, where poor outcomes are directly associated with increased TMAO levels [104]. In the multistep process of TMAO production, specific intestinal bacteria, particularly within the phyla *Firmicutes* and *Proteobacteria*, play a crucial role in converting dietary precursors into TMA. Certain families, such as *Clostridiaceae*, *Lachnospiraceae*, and *Veillonellaceae* from *Firmicutes*, and *Enterobacteriaceae* from *Proteobacteria*, were associated with high TMAO levels. Conversely, families like *Bacteroidaceae* and *Prevotellaceae* from *Bacteroidetes* are related to low TMAO levels [87]. At the genus level, TMA producers include *Clostridia*, *Shigella*, *Proteus*, and *Aerobacter*, while *Escherichia* and *Klebsiella* significantly reduce TMAO to TMA [105]. Moreover, enterotype characterisation studies show that enterotype 2 with *Prevotella* is associated with high TMAO levels, while enterotype 1, predominantly *Bacteroides*, is related to low TMAO levels [106]. At the species level, *Escherichia coli* contributes to TMA production, whereas *Enterobacter aerogenes* reduces plasma TMAO levels [107]. Additionally, TMAO and TMA are correlated with anthropometric parameters and cardiovascular health markers, including TC, LDL-C, apolipoprotein B, and tumour necrosis factor-alpha levels [40]. However, different gut microbial compositions resulted in varying amounts of TMAO. Our findings highlight that the genus *Streptococcus* contributes to elevated plasma TMAO levels, suppresses reverse cholesterol transport, and leads to atherosclerotic plaque formation. The data from Hoyles *et al.* (2018) support our observations, demonstrating that TMAO influences the growth of lactic acid bacteria, including *Streptococcus* [108].

This study highlights the complex interplay between human gut microbiota, its metabolic by-products, and CVD. The microbial diversity and specific bacterial taxa identified in this comprehensive review provide valuable insights into CVD’s potential contributors to and modulators of CVD. Moreover, the observed associations between microbial alterations, metabolite production, and cardiovascular risk factors indicate the multifaceted nature of the development and progression of CVD.

## 5. Strengths and Limitations

This research conducted an extensive investigation into the relationship between the gut microbiome and various CVDs, employing a comprehensive review of three databases. Notably, it incorporates a statistical component, which is a distinctive feature, due to the scarcity of microbiota-focused meta-analyses in the scientific literature.

However, several limitations must be considered. Discrepancies in metagenomic and metabolomic analysis methods, including various technical approaches, clustering methods, and mapping databases, create challenges regarding standardisation. Additionally, differences in population characteristics, limited causality in case-control studies, and geographical biases exacerbate the heterogeneity of the data.

In metabolomic studies, the correlation of metabolites beyond TMAO was challenging due to the requirement of data from at least three articles, and the diverse sources of metabolites (blood and faeces) complicated aggregation. Furthermore, the use of general keywords in the research strategy limited the inclusion of additional metabolites, suggesting that a more targeted approach would be beneficial.

Several factors, including the scarcity of eligible articles, reliance on aggregated data, and unavailability of raw data, have significantly hampered the meta-analysis process. The harmonisation of original data and the challenges associated with selective reporting of significant findings and tools used for data extraction from images further complicate the analysis. These issues collectively hinder the drawing of reliable correlations between the different datasets.

Despite these limitations, this study emphasises the need for further research and standardisation to explore the complex relationships between gut microbiota and CVD.

## 6. Conclusions

This meta-analysis and systematic review found distinct differences in the gut microbiota composition of patients with CVD compared with HCs. Reduced *Faecalibacterium* and increased *Streptococcus* and *Proteobacteria* were notable changes potentially linked to CVD, alongside alterations in the TMAO metabolite. Changes in biochemical parameters, such as increased FG and TG levels and decreased TC, LDL-C, and HDL-C levels, were observed in patients with CVD. Despite the strong correlations between specific bacterial taxa and CVD, further research in larger populations is needed to confirm causation. High heterogeneity and variations in reporting specifications highlight the need for standardised procedures in microbiome research. Continuous preclinical and clinical research is crucial for a comprehensive understanding of the role of gut microbiota in characterising CVD.

## Data Availability

The original contributions presented in the study are included in the article and Supplementary Materials. Further inquiries can be directed to the corresponding author.

## Supplementary Materials

The following supporting information can be downloaded at: https://www.mdpi.com/article/10.3390/biom14060731/s1, Table S1. PRISMA checklist. Table S2. PRISMA abstract checklist. Table S3. Database search strategy. Table S4. Characteristics of included studies in the systematic review and meta-analysis. Table S5. Metabolic biomarkers of the studies included in the systematic review and meta-analysis. Table S6. Information summary of all included studies that performed metagenomic analysis. Table S7. Information summary of all included studies that performed metabolomic analysis. Table S8. Quality assessment of observational cohort and cross-sectional studies included in the systematic review and meta-analysis. Table S9. Quality assessment of control-case studies included in the systematic review and meta-analysis. Figure S1. Biochemical data across different types of cardiovascular disease (CVD).
