# Supplementary material for: Unravelling the Gut Microbiome Role in Cardiovascular Disease: A Systematic Review and a Meta-Analysis
Source: Biomolecules. 2024 Jun 20;14(6):731. doi: 10.3390/biom14060731 (PMC11201797; doi:10.3390/biom14060731)
Supplement: Supplementary file 1 [file biomolecules-14-00731-s001.zip › biomolecules-3008476-supplementary.pdf]

SUPPLEMENTAL MATERIAL

Unravelling the gut microbiome role in cardiovascular disease: A systematic review and a meta-analysis

Contents

Table S1. PRISMA checklist. 2
Table S2. PRISMA abstract checklist. 5
Table S3. Database search strategy. 6
Table S4. Characteristics of included studies in the systematic review and meta-analysis. 9
Table S5. Metabolic biomarkers of the studies included in the systematic review and meta-analysis. 26
Table S6. Information summary of all included studies that performed metagenomic analysis. 29
Table S7. Information summary of all included studies that performed metabolomic analysis. 31
Table S8. Quality assessment of observational cohort and cross-sectional studies included in the systematic review and meta-analysis. 33
Table S9. Quality assessment of control-case studies included in the systematic review and meta-analysis. 34
Figure S1. Biochemical data across different types of cardiovascular disease (CVD). 36

Table S1. PRISMA checklist.

Section and Topic	Item #	Checklist item	Location where item is reported
**TITLE**			
Title	1	Identify the report as a systematic review.	Page 1, Title
**ABSTRACT**			
Abstract	2	See the PRISMA 2020 for Abstracts checklist.	Supplementary Material
**INTRODUCTION**			
Rationale	3	Describe the rationale for the review in the context of existing knowledge.	Pages 3, 4, 5
Objectives	4	Provide an explicit statement of the objective(s) or question(s) the review addresses.	Page 5, Lines 108-112
**METHODS**			
Eligibility criteria	5	Specify the inclusion and exclusion criteria for the review and how studies were grouped for the syntheses.	Page 6, Lines 125-129
Information sources	6	Specify all databases, registers, websites, organisations, reference lists and other sources searched or consulted to identify studies. Specify the date when each source was last searched or consulted.	Page 6, Lines 121-125, Table S1
Search strategy	7	Present the full search strategies for all databases, registers, and websites, including any filters and limits used.	Table S1
Selection process	8	Specify the methods used to decide whether a study met the inclusion criteria of the review, including how many reviewers screened each record and each report retrieved, whether they worked independently, and, if applicable, details of automation tools used in the process.	Page 6, Lines 131-136
Data collection process	9	Specify the methods used to collect data from reports, including how many reviewers collected data from each report, whether they worked independently, any processes for obtaining or confirming data from study investigators, and if applicable, details of automation tools used in the process.	Pages 6-7, Lines 139-162
Data items	10a	List and define all outcomes for which data were sought. Specify whether all results that were compatible with each outcome domain in each study were sought (e.g. for all measures, time points, analyses), and if not, the methods used to decide which results to collect.	Page 7, Lines 145-153
	10b	List and define all other variables for which data were sought (e.g. participant and intervention characteristics, funding sources). Describe any assumptions made about any missing or unclear information.	Page 6-7, Lines 140-144
Study risk of bias assessment	11	Specify the methods used to assess risk of bias in the included studies, including details of the tool(s) used, how many reviewers assessed each study and whether they worked independently, and if applicable, details of automation tools used in the process.	Page 8, Lines 176-181
Effect measures	12	Specify for each outcome the effect measure(s) (e.g. risk ratio, mean difference) used in the synthesis or presentation of results.	Page 7, Lines 159-160
Synthesis methods	13a	Describe the processes used to decide which studies were eligible for each synthesis (e.g. tabulating the study intervention characteristics and comparing against the planned groups for each synthesis (item #5)).	Page 7, Lines 153-158
	13b	Describe any methods required to prepare the data for presentation or synthesis, such as handling of missing summary statistics or data conversions.	Page 7, Lines 153-158

Section and Topic	Item #	Checklist item	Location where item is reported
	13c	Describe any methods used to tabulate or visually display results of individual studies and syntheses.	Page 7, Lines 153-158
	13d	Describe any methods used to synthesize results and provide a rationale for the choice(s). If meta-analysis was performed, describe the model(s), method(s) to identify the presence and extent of statistical heterogeneity, and software package(s) used.	Page 8, Lines 165-173
	13e	Describe any methods used to explore possible causes of heterogeneity among study results (e.g. subgroup analysis, meta-regression).	Not applicable
	13f	Describe any sensitivity analyses conducted to assess robustness of the synthesized results.	Page 8, Lines 165-173
Reporting bias assessment	14	Describe any methods used to assess risk of bias due to missing results in a synthesis (arising from reporting biases).	Page 8, Lines 165-173
Certainty assessment	15	Describe any methods used to assess certainty (or confidence) in the body of evidence for an outcome.	Page 8, Lines 165-173
**RESULTS**			
Study selection	16a	Describe the results of the search and selection process, from the number of records identified in the search to the number of studies included in the review, ideally using a flow diagram.	Pages 8-9, Lines 185-192, Figure 1
	16b	Cite studies that might appear to meet the inclusion criteria but which were excluded, and explain why they were excluded.	Pages 8-9, Lines 185-192, Figure 1
Study characteristics	17	Cite each included study and present its characteristics.	Table S2
Risk of bias in studies	18	Present assessments of risk of bias for each included study.	Page 9, Lines 193-195, Tables S6 and S7
Results of individual studies	19	For all outcomes, present, for each study: (a) summary statistics for each group (where appropriate) and (b) an effect estimate and its precision (e.g. confidence/credible interval), ideally using structured tables or plots.	Pages 12-14, Lines 274-319
Results of syntheses	20a	For each synthesis, briefly summarise the characteristics and risk of bias among contributing studies.	Page 9, Lines 193-203
	20b	Present results of all statistical syntheses conducted. If meta-analysis was done, present for each the summary estimate and its precision (e.g. confidence/credible interval) and measures of statistical heterogeneity. If comparing groups, describe the direction of the effect.	Pages 12-14, Lines 274-319
	20c	Present results of all investigations of possible causes of heterogeneity among study results.	Not applicable
	20d	Present results of all sensitivity analyses conducted to assess the robustness of the synthesized results.	Pages 12-14, Lines 274-319
Reporting biases	21	Present assessments of risk of bias due to missing results (arising from reporting biases) for each synthesis assessed.	Pages 12-14, Lines 274-319
Certainty of evidence	22	Present assessments of certainty (or confidence) in the body of evidence for each outcome assessed.	Pages 12-14, Lines 274-319
**DISCUSSION**			

Section and Topic	Item #	Checklist item	Location where item is reported
Discussion	23a	Provide a general interpretation of the results in the context of other evidence.	Pages 14-19, Lines 337-444
	23b	Discuss any limitations of the evidence included in the review.	Pages 19-20, Lines 453-467
	23c	Discuss any limitations of the review processes used.	Pages 19-20, Lines 453-467
	23d	Discuss implications of the results for practice, policy, and future research.	Page 20, Lines 470-480
**OTHER INFORMATION**			
Registration and protocol	24a	Provide registration information for the review, including register name and registration number, or state that the review was not registered.	Not applicable
	24b	Indicate where the review protocol can be accessed, or state that a protocol was not prepared.	Not applicable
	24c	Describe and explain any amendments to information provided at registration or in the protocol.	Not applicable
Support	25	Describe sources of financial or non-financial support for the review, and the role of the funders or sponsors in the review.	Page 21, Lines 487-489
Competing interests	26	Declare any competing interests of review authors.	Page 21, Line 502
Availability of data, code and other materials	27	Report which of the following are publicly available and where they can be found: template data collection forms; data extracted from included studies; data used for all analyses; analytic code; any other materials used in the review.	Page 20, Lines 483-484

Table S2. PRISMA abstract checklist.

Section and Topic	Item #	Checklist item	Reported (Yes/No)
**TITLE**			
Title	1	Identify the report as a systematic review.	Yes
**BACKGROUND**			
Objectives	2	Provide an explicit statement of the main objective(s) or question(s) the review addresses.	Yes
**METHODS**			
Eligibility criteria	3	Specify the inclusion and exclusion criteria for the review.	No
Information sources	4	Specify the information sources (e.g. databases, registers) used to identify studies and the date when each was last searched.	No
Risk of bias	5	Specify the methods used to assess risk of bias in the included studies.	No
Synthesis of results	6	Specify the methods used to present and synthesise results.	No
**RESULTS**			
Included studies	7	Give the total number of included studies and participants and summarise relevant characteristics of studies.	Yes
Synthesis of results	8	Present results for main outcomes, preferably indicating the number of included studies and participants for each. If meta-analysis was done, report the summary estimate and confidence/credible interval. If comparing groups, indicate the direction of the effect (i.e. which group is favoured).	Yes
**DISCUSSION**			
Limitations of evidence	9	Provide a brief summary of the limitations of the evidence included in the review (e.g. study risk of bias, inconsistency and imprecision).	No
Interpretation	10	Provide a general interpretation of the results and important implications.	Yes
**OTHER**			
Funding	11	Specify the primary source of funding for the review.	No
Registration	12	Provide the register name and registration number.	No

Table S3. Database search strategy.

PubMed	3/20/2023	Results
#1	"cardiovascular diseases"[MeSH Terms] OR ("cardiovascular"[All Fields] AND "diseases"[All Fields]) OR "cardiovascular diseases"[All Fields] OR ("cardiovascular"[All Fields] AND "disease"[All Fields]) OR "cardiovascular disease"[All Fields] OR ("heart diseases"[MeSH Terms] OR ("heart"[All Fields] AND "diseases"[All Fields]) OR "heart diseases"[All Fields] OR ("heart"[All Fields] AND "disease"[All Fields]) OR "heart disease"[All Fields]) OR (("cardiovascular system"[MeSH Terms] OR ("cardiovascular"[All Fields] AND "system"[All Fields]) OR "cardiovascular system"[All Fields] OR "cardiovascular"[All Fields] OR "cardiovasculars"[All Fields]) AND ("event"[All Fields] OR "event s"[All Fields] OR "events"[All Fields])) OR ("coronary artery disease"[MeSH Terms] OR ("coronary"[All Fields] AND "artery"[All Fields] AND "disease"[All Fields]) OR "coronary artery disease"[All Fields]) OR ("coronary disease"[MeSH Terms] OR ("coronary"[All Fields] AND "disease"[All Fields]) OR "coronary disease"[All Fields] OR ("coronary"[All Fields] AND "heart"[All Fields] AND "disease"[All Fields]) OR "coronary heart disease"[All Fields]) OR ("ischaemic heart disease"[All Fields] OR "myocardial ischemia"[MeSH Terms] OR ("myocardial"[All Fields] AND "ischemia"[All Fields]) OR "myocardial ischemia"[All Fields] OR ("ischemic"[All Fields] AND "heart"[All Fields] AND "disease"[All Fields]) OR "ischemic heart disease"[All Fields] OR "coronary artery disease"[MeSH Terms] OR ("coronary"[All Fields] AND "artery"[All Fields] AND "disease"[All Fields]) OR "coronary artery disease"[All Fields] OR ("ischemic"[All Fields] AND "heart"[All Fields] AND "disease"[All Fields])) OR ("stroke"[MeSH Terms] OR "stroke"[All Fields] OR "strokes"[All Fields] OR "stroke s"[All Fields]) OR ("stroke"[MeSH Terms] OR "stroke"[All Fields] OR ("cerebrovascular"[All Fields] AND "accident"[All Fields]) OR "cerebrovascular accident"[All Fields]) OR ("cerebrovascular disorders"[MeSH Terms] OR ("cerebrovascular"[All Fields] AND "disorders"[All Fields]) OR "cerebrovascular disorders"[All Fields] OR ("cerebrovascular"[All Fields] AND "disease"[All Fields]) OR "cerebrovascular disease"[All Fields]) OR ("myocardial infarction"[MeSH Terms] OR ("myocardial"[All Fields] AND "infarction"[All Fields]) OR "myocardial infarction"[All Fields]) OR ("myocardial infarction"[MeSH Terms] OR ("myocardial"[All Fields] AND "infarction"[All Fields]) OR "myocardial infarction"[All Fields] OR ("heart"[All Fields] AND "attack"[All Fields]) OR "heart attack"[All Fields]) OR ("peripheral arterial disease"[MeSH Terms] OR ("peripheral"[All Fields] AND "arterial"[All Fields] AND "disease"[All Fields]) OR "peripheral arterial disease"[All Fields]) OR ("peripheral vascular diseases"[MeSH Terms] OR ("peripheral"[All Fields] AND "vascular"[All Fields] AND "diseases"[All Fields]) OR "peripheral vascular diseases"[All Fields] OR ("peripheral"[All Fields] AND "vascular"[All Fields] AND "disease"[All Fields]) OR "peripheral vascular disease"[All Fields]) OR ("heart failure"[MeSH Terms] OR ("heart"[All Fields] AND "failure"[All Fields]) OR "heart failure"[All Fields]) OR ("heart failure"[MeSH Terms] OR ("heart"[All Fields] AND "failure"[All Fields]) OR "heart failure"[All Fields] OR ("cardiac"[All Fields] AND "failure"[All Fields]) OR "cardiac failure"[All Fields]) OR ("atrial fibrillation"[MeSH Terms] OR ("atrial"[All Fields] AND "fibrillation"[All Fields]) OR "atrial fibrillation"[All Fields]) OR ("atrial fibrillation"[MeSH Terms] OR ("atrial"[All Fields] AND "fibrillation"[All Fields]) OR "atrial fibrillation"[All Fields] OR ("auricular"[All Fields] AND "fibrillation"[All Fields]) OR "auricular fibrillation"[All Fields]) OR ("arrhythmia s"[All Fields] OR "arrhythmias, cardiac"[MeSH Terms] OR ("arrhythmias"[All Fields] AND "cardiac"[All Fields]) OR "cardiac arrhythmias"[All Fields] OR "arrhythmia"[All Fields] OR "arrhythmias"[All Fields]) OR ("atherosclerosis"[MeSH Terms] OR "atherosclerosis"[All Fields] OR "atheroscleroses"[All Fields]) OR ("atherosclerosis"[MeSH Terms] OR "atherosclerosis"[All Fields] OR ("atherosclerotic"[All Fields] AND "cardiovascular"[All Fields] AND "disease"[All Fields]) OR "atherosclerotic cardiovascular disease"[All Fields]) OR ("heart valve diseases"[MeSH Terms] OR ("heart"[All Fields] AND "valve"[All Fields] AND "diseases"[All Fields]) OR "heart valve diseases"[All Fields] OR ("valvular"[All Fields] AND "heart"[All Fields] AND "disease"[All Fields]) OR "valvular heart disease"[All Fields]) OR (("valve"[All Fields] OR "valve s"[All Fields] OR "valved"[All Fields] OR "valves"[All Fields] OR "valving"[All Fields]) AND ("disease"[MeSH Terms] OR "disease"[All Fields] OR "diseases"[All Fields] OR "disease s"[All Fields] OR "diseased"[All Fields]))	3,337,980

#2	"microbiota"[MeSH Terms] OR "microbiota"[All Fields] OR "microbiotas"[All Fields] OR "microbiota s"[All Fields] OR "microbiotae"[All Fields] OR ("microbiome s"[All Fields] OR "microbiomic"[All Fields] OR "microbiomics"[All Fields] OR "microbiota"[MeSH Terms] OR "microbiota"[All Fields] OR "microbiome"[All Fields] OR "microbiomes"[All Fields]) OR ("gastrointestinal microbiome"[MeSH Terms] OR ("gastrointestinal"[All Fields] AND "microbiome"[All Fields]) OR "gastrointestinal microbiome"[All Fields] OR ("gut"[All Fields] AND "microbiota"[All Fields]) OR "gut microbiota"[All Fields]) OR ("gastrointestinal microbiome"[MeSH Terms] OR ("gastrointestinal"[All Fields] AND "microbiome"[All Fields]) OR "gastrointestinal microbiome"[All Fields] OR ("intestinal"[All Fields] AND "microbiota"[All Fields]) OR "intestinal microbiota"[All Fields]) OR (("intestinalization"[All Fields] OR "intestinalized"[All Fields] OR "intestinally"[All Fields] OR "intestinals"[All Fields] OR "intestine s"[All Fields] OR "intestines"[MeSH Terms] OR "intestines"[All Fields] OR "intestinal"[All Fields] OR "intestine"[All Fields]) AND ("bacteria s"[All Fields] OR "bacteriae"[All Fields] OR "bacterias"[All Fields] OR "microbiology"[MeSH Subheading] OR "microbiology"[All Fields] OR "bacteria"[All Fields] OR "bacteria"[MeSH Terms])) OR (("bacterial"[All Fields] OR "bacterially"[All Fields] OR "bacterials"[All Fields]) AND ("communal"[All Fields] OR "communalism"[All Fields] OR "communalities"[All Fields] OR "communality"[All Fields] OR "communally"[All Fields] OR "commune"[All Fields] OR "communes"[All Fields] OR "community s"[All Fields] OR "communitys"[All Fields] OR "residence characteristics"[MeSH Terms] OR ("residence"[All Fields] AND "characteristics"[All Fields]) OR "residence characteristics"[All Fields] OR "communities"[All Fields] OR "community"[All Fields])) OR ("gastrointestinal microbiome"[MeSH Terms] OR ("gastrointestinal"[All Fields] AND "microbiome"[All Fields]) OR "gastrointestinal microbiome"[All Fields] OR ("gut"[All Fields] AND "flora"[All Fields]) OR "gut flora"[All Fields]) OR ("gastrointestinal microbiome"[MeSH Terms] OR ("gastrointestinal"[All Fields] AND "microbiome"[All Fields]) OR "gastrointestinal microbiome"[All Fields] OR ("intestinal"[All Fields] AND "flora"[All Fields]) OR "intestinal flora"[All Fields]) OR (("commensal"[All Fields] OR "commensalisms"[All Fields] OR "commensally"[All Fields] OR "commensals"[All Fields] OR "symbiosis"[MeSH Terms] OR "symbiosis"[All Fields] OR "commensalism"[All Fields]) AND ("bacteria s"[All Fields] OR "bacteriae"[All Fields] OR "bacterias"[All Fields] OR "microbiology"[MeSH Subheading] OR "microbiology"[All Fields] OR "bacteria"[All Fields] OR "bacteria"[MeSH Terms])) OR ("dysbiosis"[MeSH Terms] OR "dysbiosis"[All Fields] OR "disbiosis"[All Fields]) OR ("gastrointestinal microbiome"[MeSH Terms] OR ("gastrointestinal"[All Fields] AND "microbiome"[All Fields]) OR "gastrointestinal microbiome"[All Fields])	302,457
#	"metabolome"[MeSH Terms] OR "metabolome"[All Fields] OR "metabolomes"[All Fields] OR "metabolomics"[MeSH Terms] OR "metabolomics"[All Fields] OR "metabolomic"[All Fields] OR ("metabolic"[All Fields] OR "metabolical"[All Fields] OR "metabolically"[All Fields] OR "metabolics"[All Fields] OR "metabolism"[MeSH Terms] OR "metabolism"[All Fields] OR "metabolisms"[All Fields] OR "metabolism"[MeSH Subheading] OR "metabolities"[All Fields] OR "metabolization"[All Fields] OR "metabolize"[All Fields] OR "metabolized"[All Fields] OR "metabolizer"[All Fields] OR "metabolizers"[All Fields] OR "metabolizes"[All Fields] OR "metabolizing"[All Fields]) OR ("metabolic"[All Fields] OR "metabolical"[All Fields] OR "metabolically"[All Fields] OR "metabolics"[All Fields] OR "metabolism"[MeSH Terms] OR "metabolism"[All Fields] OR "metabolisms"[All Fields] OR "metabolism"[MeSH Subheading] OR "metabolities"[All Fields] OR "metabolization"[All Fields] OR "metabolize"[All Fields] OR "metabolized"[All Fields] OR "metabolizer"[All Fields] OR "metabolizers"[All Fields] OR "metabolizes"[All Fields] OR "metabolizing"[All Fields]) OR ("biomarker s"[All Fields] OR "biomarkers"[MeSH Terms] OR "biomarkers"[All Fields] OR "biomarker"[All Fields]) OR ("metabolite"[All Fields] OR "metabolite s"[All Fields] OR "metabolites"[All Fields]) OR ("gut-derived"[All Fields] AND ("metabolite"[All Fields] OR "metabolite s"[All Fields] OR "metabolites"[All Fields])) OR ("microbiota-derived"[All Fields] AND ("metabolite"[All Fields] OR "metabolite s"[All Fields] OR "metabolites"[All Fields]))	9,531,116
#4	#1 AND #2 AND #3	6,391
#5	#4 NOT (((((("case reports"[Publication Type]) OR ("comment"[Publication Type])) OR ("editorial"[Publication Type])) OR ("letter"[Publication Type])) OR ("meta analysis"[Publication Type])) OR ("review"[Publication Type])) OR ("systematic review"[Publication Type])	3,797
#6	#5 AND (humans[Filter])	2,014
**Web of Science**	3/20/2023	**Results**
#1	((((((((((((((((((((cardiovascular disease) OR (heart disease)) OR (cardiovascular events)) OR (coronary artery disease)) OR (coronary heart disease)) OR (ischemic heart disease)) OR (stroke)) OR (cerebrovascular accident)) OR (cerebrovascular disease)) OR (myocardial infarction)) OR (heart attack)) OR (peripheral arterial disease)) OR (peripheral vascular disease)) OR (heart failure)) OR (cardiac failure)) OR (atrial fibrillation)) OR (auricular fibrillation)) OR (arrhythmia)) OR (atherosclerosis)) OR (atherosclerotic cardiovascular disease)) OR (valvular heart disease)) OR (valve disease) [All fields]	2,331,155

#2	((((((((((microbiota) OR (microbiome)) OR (gut microbiota)) OR (intestinal microbiota)) OR (intestinal bacteria)) OR (bacterial communities)) OR (gut flora)) OR (intestinal flora)) OR (commensal bacteria)) OR (dysbiosis)) OR (gastrointestinal microbiome) [All fields]	265,993
#3	((((((metabolome) OR (metabolism)) OR (metabolic)) OR (biomarker)) OR (metabolites)) OR (gut-derived metabolites)) OR (microbiota-derived metabolites) [All fields]	2,476,220
#4	#1 AND #2 AND #3	5,188
#5	#4 NOT [Document Type] Book, Book chapter, Editorial Material, Letter, Review	3,123
#6	#5 AND [Topic] Human	937
**Scopus**	3/20/2023	**Results**
#1	TITLE-ABS-KEY((((((((((((((((((((cardiovascular disease) OR (heart disease)) OR (cardiovascular events)) OR (coronary artery disease)) OR (coronary heart disease)) OR (ischemic heart disease)) OR (stroke)) OR (cerebrovascular accident)) OR (cerebrovascular disease)) OR (myocardial infarction)) OR (heart attack)) OR (peripheral arterial disease)) OR (peripheral vascular disease)) OR (heart failure)) OR (cardiac failure)) OR (atrial fibrillation)) OR (auricular fibrillation)) OR (arrhythmia)) OR (atherosclerosis)) OR (atherosclerotic cardiovascular disease)) OR (valvular heart disease)) OR (valve disease))	2,810,439
#2	TITLE-ABS-KEY ((((((((((microbiota) OR (microbiome)) OR (gut AND microbiota)) OR (intestinal AND microbiota)) OR (intestinal AND bacteria)) OR (bacterial AND communities)) OR (gut AND flora)) OR (intestinal AND flora)) OR (commensal AND bacteria)) OR (dysbiosis)) OR (gastrointestinal AND microbiome)	326,604
#3	TITLE-ABS-KEY ((((((metabolome) OR (metabolism)) OR (metabolic)) OR (biomarker)) OR (metabolites)) OR (gut-derived AND metabolites)) OR (microbiota-derived AND metabolites)	5,193,133
#4	#1 AND #2 AND #3	5,545
#5	#4 AND (EXCLUDE (DOCTYPE,"re") OR EXCLUDE (DOCTYPE,"ch") OR EXCLUDE (DOCTYPE,"ed") OR EXCLUDE (DOCTYPE,"no") OR EXCLUDE (DOCTYPE,"sh") OR EXCLUDE (DOCTYPE,"cp") OR EXCLUDE (DOCTYPE,"le") OR EXCLUDE (DOCTYPE,"er") OR EXCLUDE (DOCTYPE,"bk") OR EXCLUDE (DOCTYPE,"tb") OR EXCLUDE (DOCTYPE,"dp") OR EXCLUDE (DOCTYPE,"cr"))	2,636
#6	#5 AND (LIMIT-TO (EXACTKEYWORD,"Human"))	1,756

Table S4. Characteristics of included studies in the systematic review and meta-analysis.

BMI, Body mass index; CVD, Cardiovascular disease; HC, Healthy control; NR, Not reported; SD, Standard deviation.

Study	Country	Sample size		Age (mean ± SD)		Male (n (%))		BMI (mean ± SD)		Description of patients	Description of controls	Exclusion criteria
		CVD	HC	CVD	HC	CVD	HC	CVD	HC			
**Acute Coronary Syndrome**												
Alhmoud et al. 2019	United States of America	19	19	54.4 ± 2.2	49.0 ± 1.6	14 (74%)	13 (68%)	28.3 ± 1.9	24.3 ± 0.4	Patients admitted with a diagnosis of acute coronary syndrome described by the American Heart Association, including ST-elevation myocardial infarction and non-ST segment elevation myocardial infarction.	Age and sex-matched controls	Patients: less than 19 years, pregnancy, abnormal kidney function, heart failure, intensive care unit level of care. Controls: hypertension, diabetes or dyslipidemia, prior heart attacks or strokes, tobacco use, antibiotic use within three months prior to enrollment.
Dai et al. 2020	China	254	247	63.07 ± 11.02	62.77 ± 10.69	217 (85.4%)	213 (86.2%)	24.73 ± 3.53	25.41 ± 3.08	Patients with documented ≥ 50% stenosis of at least one epicardial coronary artery during the coronary angiography	Non-acute coronary syndrome participants who underwent coronary angiography with a normal coronary artery	NR
Gao et al. 2020	China	60	30	54.92 ± 8.51	52.65 2 ± 8.79	48 (80%)	22 (73.3%)	25.48 ± 2.42	24.39 ± 2.86	Patients: cardiac troponin I/T> the upper limit of the normal reference value or creatine kinase isoenzyme>the upper limit of the normal reference value; electrocardiogram with ST-segment elevation on 2 or more adjacent leads; persistent ischemic chest pain, abnormal segmental wall motion upon electrocardiogram, and abnormal coronary angiography.	Healthy volunteers recruited from the hospital health examination center.	All: history of organic digestive system or digestive tract surgery; history of stroke, hypertension, diabetes, kidney disease or respiratory diseases; history of smoking or alcohol abuse; infection within 1 month of the study or using a probiotic, antacid, antibiotic, or preparation.

Kwun, J. et al 2020	Korea	22	20	59.4 ± 11.4	59.7 ± 11.8	20 (90.9%)	18 (90.0%)	NR	NR	Patients with electrocardiogram criteria of at least 1 mm in 2 or more standard leads or at least 2 mm in 2 or more contiguous precordial leads of ST-segment elevation or new left bundle-branch block; and angiographically proven coronary thrombi.	Control individuals enrolled voluntarily through poster advertisements among those undergoing routine health checks.	Patients: other identifiable etiologies of coronary thrombi (e.g., coronary vasospasm and systemic thromboembolism); and evident active infection during admission.
Han et al. 2021	China	30	30	62.6 ± 9.02	60.0 ± 9.64	18 (60%)	10 (33%)	25.4 ± 3.33	24.9 ± 3.08	Patients' diagnosis in symptoms of ischemia, cardiac laboratory biomarker data, electrocardiogram results and invasive coronary angiograms or coronary computed tomography angiography.	Healthy individuals with no ischemic symptoms, normal electrocardiogram, and coronary stenosis of <25% as assessed by invasive coronary angiograms or coronary computed tomography angiography.	Patients: antacids, probiotics, antibiotics, or antimicrobial agents within 30 days before sample collection, organic digestive system disease, and gastrointestinal surgery.
Chiu et al. 2022	China	19	25	65.15 ± 11.53	73.20 ± 9.06	NR	NR	26.4	24.02	Patients older than 20 years and with suspected acute coronary syndrome	NR	Patients: without the final diagnosis of ST-elevation myocardial infarction after coronary artery intervention.<br>All: prior gastrointestinal surgery, ongoing infection, antibiotics, inflammatory bowel disease, auto-immune diseases, malignancy, history of stroke, renal failure, hepatic diseases, digestive diseases, smoking and alcohol, antacids or probiotics within 3 months of fecal sample collection.
Liu, C. et al. 2022	China	117	78	62.4 ± 12.3	62.5 ± 9.6	113 (88%)	63 (80.8%)	24.8 ± 3.2	25.6 ± 2.5	Patients with circulating level of cardiac troponin above the 99th percentile upper reference limit and diagnosed as obstructive atherosclerotic myocardial infarction	Individuals with no stenosis or only a few minor irregularities (<30% stenosis).	All: not stool samples, history of physician diagnosed diabetes, acute myocardial injury of non-ischemic origin, and any prior coronary artery disease.

										by coronary angiography.		
Qian et al. 2022	China	20	20	65.8 ± 63.06	63.6 ± 72.45	12 (60%)	9 (45%)	22.81 ± 11.18	21.67 ± 10.73	Acute myocardial infarction patients were diagnosed with ST-segment elevation myocardial infarction according to electrocardiographic examination and laboratory examination of myocardial enzymes.	Healthy controls were from the patients' families or close relatives with similar age and no other diseases.	Patients: digestive system-related diseases in the past 3 years, history of surgery, history of smoking, alcoholism, and various metabolic diseases.
Tang et al. 2022	China	51	55	64.0 ± 17.04	61.33 ± 16.30	39 (76.5%)	26 (47.4%)	NR	NR	Patients who had a detection of cardiac troponin I > 0.5 μg/L, with at least one (a) symptom of myocardial ischemia; (b) new ischemic electrocardiogram changes; (c) development of pathological Q waves; (d) identification of a coronary thrombus by angiography.	The healthy individuals exhibited normal biochemical marker levels (protein, a fasting blood glucose, and lipid-associated markers).	NR
**Atherosclerosis**												
Karlsson et al. 2012	Sweden	12	13	67.6 ± 8.6	70.5 ± 0.5	9 (75%)	10 (76.9%)	25.8 ± 2.4	23.7± 2.9	Patients with severely stenotic plaques in the carotid artery with ipsilateral manifestations of emboli to either the brain, as minor brain infarction or transient ischemic symptoms, or to the retinal artery.	Age- and sex-matched groups with no cardiovascular health problems recruited from two population-based cohorts.	NR
Jie et al. 2017	China	218	187	61 ± 9.74	60 ± 9.77	161 (73.9%)	75 (40.1%)	25 ± 6.59	24 ± 3.49	Patients with clinical presentations of stable angina, unstable angina, or acute myocardial infarction were diagnosed by coronary angiography. Individuals with ≥50% stenosis in single or multiple vessels were included.	Healthy individuals enrolled with free of clinically evident atherosclerotic cardiovascular disease symptoms during the medical examination.	Patients: ongoing infectious diseases, cancer, renal, or hepatic failure, peripheral neuropathy, stroke, use of antibiotics within 1 month of sample collection.<br>Controls: peripheral artery disease, known coronary artery disease or myocardial infarction,

												cardiomyopathy, renal failure, peripheral neuropathy, systemic disease, and stroke.
Ji, L. et al. 2021	China	32	32	64.5 ± 6.7	66.2 ± 4.8	28 (87.5%)	28 (87.5%)	24.7 ± 2.7	23.2 ± 2.2	Patients diagnosed carotid atherosclerosis by ultrasound or computed tomography angiography, with age > 45 years	NR	All: Antibiotic and probiotic usage within 6 months, history of gastrointestinal diseases, history of abdominal surgery, major dietary change 1 week before sample collection.
Stø et al. 2021	Norway	43	38	72.5 ± 6.3	67.2 ± 7.9	19 (44.2%)	11 (28.9%)	25.5 ± 3.8	24.8 ± 3.6	Patients with severe atherosclerosis; defined as moderate (50–69%) or severe (≥70%) carotid stenosis; consecutively recruited at Oslo University Hospital.	Healthy control subjects were recruited from the same area of Norway as the patients, with twelve of them being the patients' spouses.	Patients: known immunodeficiency or cancer.
**Atrial Fibrillation**												
Zuo, K. Li, J. Li. K. et al. 2019	China	50	50	64.35 ± 10.56	54.33 ± 5.18	32 (64%)	41 (82%)	26.30 ± 4.33	25.06 ± 3.58	Patients with nonvalvular atrial fibrillation enrolled from Beijing Chaoyang Hospital	Individuals enrolled from Kailuan cohort who received biennial medical examination in Kailuan General Hospital.	Patients: history of heart failure, coronary heart disease, structural heart disease, comorbidities (inflammatory bowel diseases, irritable bowel syndrome, autoimmune diseases, liver diseases, renal diseases, or cancer), or use of antibiotics or probiotics in the past 1 month.
Buttner et al. 2020	Germany	45	20	65 ± 10	65 ± 9	18 (40%)	11 (55%)	30 ± 4.5	30 ± 4.9	Patients with symptomatic non-valvular atrial fibrillation undergoing first atrial fibrillation radiofrequency	Patients from the cardiology outpatient clinic at the Heart Center Leipzing	Patients: pregnancy, cancer, acute or systemic inflammatory diseases, renal disfunction or dialysis
Zuo, K. Yin, X. et al. 2020	China	20	50	67.33 ± 8.52	54.33 ± 5.19	NR	41 (82%)	27.84 ± 5.06	25.06 ± 3.58	Patients with nonvalvular atrial fibrillation enrolled from Beijing Chaoyang Hospital	Individuals enrolled from Kailuan cohort who received biennial medical examination in Kailuan General Hospital	Patients: history of heart failure, coronary heart disease, structural heart disease, comorbidities (inflammatory bowel diseases, irritable bowel syndrome, autoimmune diseases, liver diseases, renal diseases, or cancer), or use of

												antibiotics or probiotics in the past 1 month.
Tabata et al. 2021	Japan	34	66	65.6 ± 1.4	65.3 ± 0.9	26 (76.5%)	50 (77.0%)	24.87 ± 3.41	23.96 ± 3.93	Patients admitted to Kobe University Hospital for catheter ablation against atrial fibrillation.	Individuals extracted from a healthy Japanese cohort	Patients: hepatic diseases, renal failure, collagen disease, malignancy, active infectious diseases, inflammatory intestinal diseases, moderate and severe organic heart disease, and antibiotic or steroid treatment within 2 weeks prior to admission.
Huang, K. et al. 2022	China	36	30	55.75 ± 12.35	49.17 ± 12.59	25 (69.4%)	22 (73.3%)	24.11 ± 3.16	25.25 ± 3.03	Patients with nonvalvular atrial fibrillation hospitalized in Nanfang Hospital of Southern Medical University	Individuals matched with the experimental group for sex, age, body mass index, hypertension, and diabetes history	Patients: history of heart failure, coronary heart disease, structural heart disease, comorbidities (inflammatory bowel diseases, irritable bowel syndrome, autoimmune diseases, liver diseases, renal diseases, or cancer) or the use of antibiotics or probiotics in the past month
**Coronary Artery Disease**												
Yoshida et al. 2018	Japan	30	30	63.6 ± 7.2	62.9 ± 6.8	27 (90%)	23 (77%)	25.1 ± 2.8	24.8 ± 4.1	Patients with stable angina pectoris and old myocardial infarction with preserved left ventricular ejection fraction (>40%) who underwent percutaneous coronary intervention or coronary artery bypass graft surgery ≥6 months before the study.	Individuals without coronary artery disease with coronary risk factors such as hypertension, diabetes mellitus, or dyslipidemia, but without present or history of coronary or other vascular diseases.	Patients: acute coronary syndrome, systemic disease, including hepatic disease, renal disease, collagen disease, or malignancy, antibiotics treatment.
Zhu et al. 2018	China	70	98	63.63	60.01	30 (42.9%)	41 (41.8%)	24.34	23.83	Patients recruited in Shanghai Tenth People's Hospital and confirmed by coronary angiography, including only individuals with various degrees of haemato-stenosis.	Healthy individuals enrolled via a community survey.	Patients: cancer, infectious diseases such as inflammatory bowel disease, diarrhea, antibiotic exposure, or probiotic consumption within 1 month before sample collection. Controls: individuals with coronary artery disease, myocardial

												infarction, coronary artery bypass surgery history, or any other cardiac-related disease or systemic disease.
Zhang, Y. et al. 2019	China	24	23	63.50 ± 7.70	64.04 ± 7.30	17 (70.8%)	16 (69.6%)	24.46 ± 5.80	24.83 ± 4.32	Patients admitted to the Department of Gastroenterology or Cardiology in Peking University People's Hospital	Healthy controls free of non-alcoholic fatty liver disease, coronary artery disease and none clinically coronary artery disease evidence such as angina and abnormal electrocardiographic.	All: viral hepatitis, autoimmune liver disease and alcoholic hepatitis, chronic gastrointestinal disease and previous abdominal surgery, heart failure, pregnancy women or abortion, antibiotics for nearly 2 weeks, drinking alcohol, spicy food, yogurt and probiotics for nearly 1 week.
Liu, F. et al. 2020	China	18	23	53.3 ± 6.7	41.5 ± 9.6	18 (100%)	10 (43.5%)	26.5 ± 5.4	25.2 ± 3.7	Coronary heart disease group: Tibetan patients with coronary artery stenosis >50%	Health individuals were Tibetan native residents, had similar living habits, and were required to complete the same questionnaires as the patients, and was investigated the electrocardiogram and biochemical indexes in the blood.	Patients: acute and chronic inflammatory diseases, including digestive system diseases, tumors, renal failure, and other serious diseases. Controls: history of cardiovascular disease, diabetes, and hyperlipidemia treatment
Zheng, YY. et al. 2020	China	152	105	57.94 ± 9.70	57.69 ± 10.86	112 (73.7%)	51 (48.6%)	NR	NR	Patients with ≥50% stenosis in at least one main coronary artery according to coronary angiography,	Patients without artery stenosis act as controls	Patients: heart failure, structural heart disease, pulmonary heart disease, history of antibiotic use within 1 month, and serious dysfunction of liver or kidney
Nguyen, Chong et al. 2021	France	45	35	66 ± 1.3	57 ± 2.3	33 (73%)	17 (49%)	25.9 ± 0.5	26 ± 0.75	NR	NR	All: non-fasting state or treatment with statins or fibrates, corticosteroids, anti-human immunodeficiency virus agents, antimicrobials in the past 3 months, or liver disease or cancer, or recent cardiac arrest, or cholecystectomy.
Sawicka-Smiarowska et al. 2021	Poland	169	166	64.1 ± 7.7	62.4 ± 10.5	124 (73%)	108 (65%)	30.52 ± 5.3	28.46± 4.73	Aged 30-79 years, with hospitalization 12–18 months before the evaluation for elective percutaneous	Randomly selected from the Bialystok population with sex and age matched to	Patients: history of intestinal acute or chronic disease, active cancer, aged below 30 and over 80, lack of

										coronary intervention and acute coronary syndromes.	coronary artery disease group.	informed consent; Controls: angiographic confirmation of coronary artery disease, history of acute coronary syndrome, typical angina.
Tian et al. 2021	China	71	55	62.26 ± 8.91	55.56 ± 9.48	49 (69%)	27 (49.1%)	24.30 ± 2.83	24.09 ± 3.53	Patients with coronary artery disease hospitalized for coronary angiography	Healthy volunteers consecutively recruited	All: gastrointestinal diseases, malignant tumors, autoimmune disorders, infectious diseases, renal dysfunction, a history of gastrointestinal surgery in the previous year or were administered antibiotics for more than 3 days in the previous 3 months.
Cheng et al. 2022	Tibet	14	18	54 ± 2.84	42 ± 2.18	13 (92.9%)	9 (50%)	29.54 ± 3.93	26.11 ± 0.91	Coronary heart disease group: Patients with coronary artery stenosis of >50%	Tibetan native residents, healthy with no history of coronary heart disease, diabetes, and treated hyperlipidemia, and with similar living habits.	All: subjects with non-coronary heart disease, acute and chronic inflammatory diseases, including those in the digestive system, tumors, renal failure and other serious diseases affecting the gut microbiota.
Choroszy et al. 2022	Poland	15	15	67.2 ± 9.0	57.0 ± 11.1	11 (73.3%)	5 (33.3%)	29.4 ± 5.05	25.95 ± 4.21	Coronary artery disease was confirmed by coronary angiography, and patients with ≥50% stenosis in single or multiple vessels were included	NR	All: renal disease defined as an abnormal creatinine serum level (>2 mg/dL), malignancy, ongoing infectious disease, hepatic disease, and use of antibiotics within four weeks before sample collection.
Li, W et al. 2022	China	19	19	62.63 ± 7.08	57.74 ± 9.48	8 (42%)	9 (47%)	27.19 ± 3.03	22.91 ± 2.13	Patients aged 45 to 79 years; diagnosed with coronary heart disease by physicians in a secondary hospital; written informed consent and willingness to participate in this study.	Healthy controls had no history of chronic diseases, taking any medication, or severe lifestyle that might disrupt gut microbiota.	All: other diseases (e.g., cerebrovascular diseases, metabolism diseases, gastrointestinal diseases, respiratory diseases, immunity diseases, cancers, or disease-related complications), antibiotic therapy or probiotics within three months, or cognitive impairment and cannot cooperate

Liu, L. et al. 2022	China	10	10	65.70 ± 6.67	65.50 ± 8.95	7 (70%)	7 (70%)	22.55 ± 2.62	22.91 ± 1.52	Unstable angina pectoris patients confirmed by a cardiologist and defined by chest pain at rest or angina equivalent, transient ST-T segment depression but without increased cardiac enzymes in the serum.	NR	Patients: history of chronic disease, congenital heart diseases, aortic aneurysm, cardiac valve diseases, connective tissue diseases, and cancer; history of organic digestive system or digestive tract surgery; history of smoking or alcohol abuse; use of antibiotic within 1 month
Liu, X. et al. 2022	China	133	133	64.6 ± 9.6	65.2 ± 9.1	51 (38.3%)	51 (38.3%)	25.0 ± 3.1	23.9 ± 3.1	Patients, who were diagnosed with unstable angina for the first time without a prior history of CHD following the international guidelines	NR	Patients: history of peptic ulcer, inflammatory bowel disease, gastrointestinal tumor or gastrointestinal surgery, or took any antibiotics, probiotics or prokinetic agents within three months before fecal sample collection. Controls: history of coronary heart disease, stroke, peripheral arterial disease, congenital heart disease, acute infection, cancer, or abnormal electrocardiogram with ST-segment changes.
Tian et al. 2022	China	51	14	62.49 ± 10.15	54.71 ± 8.09	31 (60.8%)	6 (42.9%)	25.83 ± 3.13	23.78 ± 2.50	Patients who underwent coronary angiography at the Peking Union Medical College Hospital diagnosed with coronary artery disease based on ≥ 50% stenosis in at least one main coronary artery.	Individuals enrolled from Beijing residents who exhibited negative results upon coronary artery computed tomography or coronary angiography examination and had no history of known cardiovascular diseases, or coronary artery disease related clinical signs and symptoms.	Patients: gastrointestinal diseases, malignant tumors, autoimmune disorders, infectious diseases, renal dysfunction (eGFR 30 ml/min/1.73 m2), folic acid and other drugs supplementation within two weeks, a history of gastrointestinal surgery in the previous year or were administered antibiotics for more than 3 days in the previous 3 months.
Yu et al. 2022	China	36	9	59.97 ± 11.33	61.56 ± 8.59	25 (69%)	5 (55.6%)	23.68 ± 3.32	22.84 ± 3.06	Patients who had undergone coronary angiography at the Affiliated Hospital of Southwest Medical University, with more	Subjects with no coronary plaques and smooth intima	Patients: gastrointestinal disease, history of gastrointestinal surgery in one year, or gut microbiome preparations or

										than 75% stenosis of any of coronary arteries or branches.		antibiotics in the past 1 month
Zhong et al. 2022	China	42	46	73.12 ± 3.79	58.17± 4.30	15 (35.7%)	20 (43.5%)	24.25 ± 1.66	23.51 ± 1.33	Patients aged between 40 and 85 years; with history of angina pectoris; myocardial ischemia, at rest, detected by electrocardiogram, or positive exercise stress test, or greater than 50% stenosis in at least 1 main branch detected by coronary angiography/coronary computed tomography.	NR	Patients: history of angina pectoris caused by heart valve disease, coronary artery embolism, cardiomyopathy; combined with heart diseases, autonomic dysfunction, obvious anemia, obstructive emphysema, or electrolyte disorder; medication of digitalis.
**Heart Failure**												
Trøseid et al. 2015	Norway	155	33	57 ± 11	59.8 ± 2.9	128 (82.6%)	NR	26.7 ± 5.6	NR	Patients with stable heart failure for > 6 months, in New York Heart Association functional class II–IV.	Healthy individuals included based on disease history and clinical examination, matched for sex and age.	Patients: acute coronary syndromes during the previous 6 months or significant concomitant diseases such as infection, malignancy, or autoimmune disease.
Cui et al. 2018	China	53	41	58.08 ± 13.30	53.73 ± 5.94	44 (83%)	32 (78%)	24.42 ± 4.53	25.24 ± 3.32	Patients with age >18 years old, medical history of chronic heart failure either from ischemic or dilated cardiomyopathy > 6 months, the New York Heart Association functional classification II to IV and left ventricular ejection fraction ≤40%.	Healthy individuals enrolled from Kailuan cohort who received biennial medical examination in Kailuan General Hospital.	Patients: history of acute coronary syndrome in the last 6 months, comorbidities (inflammatory bowel diseases, irritable bowel syndrome, autoimmune diseases, liver diseases, renal diseases or cancer), or use of antibiotics or probiotics in the last 1 month.
Hayashi et al. 2018	Japan	22	11	72 ± 18	72 ± 7	14 (64%)	6 (55%)	25.8 ± 7.1	24.4 ± 3.1	Patients prospectively enrolled and admitted to Kobe University Hospital for de novo acute decompensated heart failure or acute worsening of chronic heart failure.	Individuals recruited based on age-, sex-, and comorbidity-matched controls with heart failure risk factors but no history of heart failure who were hospitalized at Kobe University Hospital.	Patients: acute coronary syndrome, renal failure, active infectious diseases, malignancy, autoimmune disorders, inflammatory or malabsorptive intestinal diseases, history of enterectomy, hepatic diseases, antibiotic or steroid treatment within 1 month before

												admission and during hospitalization.
Katsimichas et al. 2018	Japan	28	19	51 ± 10	36 ± 6	21 (75%)	16 (84%)	21.7 ± 3.4	21.8 ± 1.9	Consecutive recruitment of patients with non-ischemic heart failure with reduced ejection fraction admitted to Osaka University Hospital.	Healthy volunteers	All: age <18 or >70 years, systemic use of oral or intravenous antibiotics within 4 weeks, topical skin use of antibiotic-containing creams/ointments within 1 week, ingestion of probiotic-containing preparations within 4 weeks before stool sampling day, major change in dietary habits, obesity, chronic kidney disease stages 4/5, intestinal disorders, intestinal surgery in the past 5 years
Dong et al. 2020	China	61	57	63.23 ± 13.83	61.91 ± 9.58	25 (41.0%)	23 (40.3%)	NR	NR	Patients having chronic heart failure with preserved ejection fraction	NR	Patients: left ventricular ejection fraction of less than 40%, severe hepatic disease, simultaneous infection, and/or renal dysfunction diseases.
Emoto et al. 2021	Japan	22	11	73.5 ± 17.04	72.67 ± 9.26	14 (63%)	6 (54%)	NR	NR	Patients recruited at Kobe University Hospital	Individuals with age-, sex-, and comorbidity-matched control with heart failure risk factors but no history of heart failure	NR
Wang et al. 2021	China	25	25	65 ± 3.17	65 ± 3.07	14 (56%)	13 (52%)	29.7 ± 1.44	29.1 ± 1.33	Patients clinically stable receiving constant medication for at least 4 weeks prior to the evaluation	Healthy subjects did not take any drugs	All: subjects with infection, rheumatoid arthritis, renal failure, major valvular heart disease, intestinal disease, cancer, or autoimmune disease.
Sun et al. 2022	China	29	30	60.69 ± 11.67	60.0 ± 9.64	24 (83%)	10 (33%)	20.4 ± 3.47	24.9 ± 3.08	Patients with multiple stages of HF progression, as defined by New York Heart Association Class III-IV.	Asymptomatic persons undergoing physical examinations	All: subjects that received antacids, probiotics, antibiotics, or antimicrobial agents within 30 days before sample collection, with organic disease of the digestive system and gastrointestinal surgery.

Peng et al. 2023	China	33	15	71.76 ± 97.93	67.67 ± 9.76	24 (72%)	8 (53%)	24.24 ± 2.81	23.52 ± 3.12	Patients aged ≥ 65 years old with similar diet and environmental conditions in the Second Xiangya Hospital of Central South University.	NR	Patients: recurrent diarrhea or constipation, unusual dietary habits (vegetarians), edema, with tumors, diabetes, intestinal inflammation, irritable bowel syndrome, history of intestinal surgery, antibiotics or probiotics treatment within 1 month.
**Hypertension**												
Li, J. et al. 2017	China	99	41	53.6 ± 5.5	53.7 ± 5.9	92 (92.9%)	32 (78.1%)	26.0 ± 3.5	25.2 ± 3.3	Patients recruited from a cohort study among employees of the Kailuan Group Corporation with systolic blood pressure≤140 mmHg, or diastolic blood pressure≤90 mmHg	Healthy individuals recruited from a cohort study among employees of the Kailuan Group Corporation with systolic blood pressure ≤125 mmHg, or diastolic blood pressure ≤80 mmHg	Patients: cancer, heart failure, renal failure, smoking, stroke, and peripheral artery disease, antihypertensive treatment. Controls: history of diabetes mellitus or hypercholesterolemia. All: antibiotics or probiotics within the last 8 weeks.
Yan et al. 2017	China	60	60	57.0 ± 9.6	56.0 ± 8.6	35 (58.3%)	32 (53.3%)	23.5 ± 2.9	23.4 ± 2.6	Blood pressure ≥140/90 mmHg	Gender-, age-, and body weight matched healthy controls with blood pressure ≤ 120/80 mm Hg	Patients: symptoms of respiratory infection or digestive tract disease, antibiotics, or anti-inflammatory agents within 2 months before sampling. Controls: hypertension or severe cardiovascular diseases history in previous 5 years
Zuo, K. Li, J. et al. 2019	China	34	15	53.83 ± 6.30	56.67 ± 5.93	31 (91.2%)	11 (73.3%)	25.34 ± 4.05	25.90 ± 2.54	Patients with hypertension diagnosis >140 mmHg for systolic blood pressure and >90 mmHg for diastolic blood pressure.	Healthy individuals as matched controls enrolled from the Kailuan cohort.	All: history of heart failure, coronary heart disease, arrhythmia, structural heart disease, comorbidities, (inflammatory bowel diseases, irritable bowel syndrome, autoimmune diseases, liver diseases, renal diseases, or cancer) or use of antibiotics or probiotics in the previous month

Calderon-Perez et al. 2020	Spain	29	32	53.7 ± 9.6	41.1 ± 9.1	19 (65.5%)	16 (50%)	26.2 ± 2.5	23.8 ± 2.7	Subjects recruited by using tableaux advertisements in the Hospital Universitari Sant Joan and from databases of volunteers who have previously participated in studies.<br>Patients with grade I hypertension, systolic blood pressure 140-159 mmHg and without significant complications, not using antihypertensive treatment.	Controls with optimal systolic blood pressure<120mmHg	All: body mass index≥30 kg/m2; fasting glucose > 126mg/dL; low-density lipoprotein-cholesterol > 190mg/dL; triglycerides > 350mg/dL, smoking, anemia, or intestinal disorders; antibiotics or probiotics within the last three months and vegetarian diet.
Takagi et al. 2020	Japan	97	54	64.33 ± 37.04	56.5 ± 53.33	49 (50.5%)	21 (38.9%)	NR	NR	Patients with systolic blood pressure ≥140 mmHg, diastolic blood pressure ≥90 mmHg, or current use of antihypertensive drugs.	NR	Controls: gastrointestinal inflammatory diseases, medication of antibiotics, corticosteroids, immunosuppressants or acid-suppressing agents within the past 3 months, history of underlying malignant disease, metabolic, respiratory, cardiologic, renal, hepatic, hematologic, neurologic, or psychiatric dysfunction and medications that affect intestinal motility, prebiotics, or probiotics, and pregnant and lactating women.
Nakai et al. 2021	Australia	23	47	60.3 ± 6.6	59.2 ± 7.7	15 (65%)	16 (34%)	26.0 ± 2.6	24.9 ± 3.0	Patients recruited from the community in metropolitan Baker Institute and Alfred Hospital (Melbourne) and regional (Shepparton) areas.	Individuals recruited in the same conditions as patients	All: gastrointestinal disease (history of intestinal surgery, inflammatory bowel disease, celiac disease, lactose intolerance, chronic pancreatitis, or other malabsorption disorder), diabetes type 1 and 2, chronic kidney disease, probiotics or antibiotics use in the past three months.

**Stroke**												
Yin et al. 2015	China	322	231	61 ± 19	56 ± 11	220 (68.3%)	130 (56.3%)	23.62 ± 3.90	25.16 ± 4.25	Patients with large-artery atherosclerotic ischemic stroke and transient ischemic attack recruited from the Department of Neurology.	Asymptomatic individuals undergoing physical examinations recruited at Health Examination Center of Nanfang Hospital.	Patients: <18 years, with radiological evidence of intracerebral hemorrhage, an apparent cause of stroke or transient ischemic attack unrelated to atherosclerosis, severe comorbid illness, an unstable medical condition, or consumption of probiotics or antibiotics within one month before admission.
Ji et al. 2017	China	10	10	NR	NR	6 (60%)	5 (50%)	NR	NR	Patients recruited from Tianjin Huanhu Hospital and diagnosed by skull computed tomography examination.	Healthy volunteers	All: antibiotics in the week before the specimen collection. Controls: metabolic, cardiovascular, or cerebrovascular diseases or cancer.
Nie, J. et al. 2018	China	622	622	62.2 ± 7.3	62.2 ± 7.3	292 (46.9%)	292 (46.9%)	25.3 ± 3.8	24.8 ± 3.4	Stroke patients included from China Stroke Primary Prevention Trial study	Randomly chosen from the baseline study participants who did not develop stroke during the follow-up, matched for age (±1 year), sex, treatment group, and study site with the cases on a 1:1 ratio.	Patients: history of physician-diagnosed stroke, myocardial infarction, heart failure, coronary revascularization, or congenital heart disease. Controls: missing trimethylamine N-oxide data
Wang et al. 2018	China	10	10	NR	NR	NR	NR	NR	NR	Patients with cerebral infarction diagnosed and treated in Tianjin Huanhu Hospital	Healthy individuals enrolled from in Tianjin Huanhu Hospital	Controls: metabolic disease, cerebrovascular disease, malignant tumors, or other conditions
Li, N. et al. 2019	China	30	30	60.47 ± 10.57	64.17 ± 12.67	21 (70%)	18 (60%)	NR	NR	Patients recruited from Qilu Hospital and diagnosed by skull computed tomography examination	Volunteers	All: antibiotics or probiotic before 4 weeks prior to the specimen collection Controls: metabolic, cardiovascular, or cerebrovascular diseases or cancer
Li, H. et al. 2020	China	79	98	66.61 ± 12.07	64.01 ± 10.44	50 (63.3%)	57 (58.2%)	24.22 ± 2.34	23.83 ± 2.11	Patients underwent computed tomography and were diagnosed by a neurologist.	Healthy volunteers	Patients: cancer, infection, history of intestinal disease, exposure to antibiotics or probiotics within 1 month before sample collection, neurological disorders,

												neuropsychiatric diseases, or a history of craniocerebral surgery
Schneider et al. 2020	Germany	193	100	69 ± 6	65.67 ± 13.33	122 (63%)	53 (53%)	NR	NR	Patients admitted to the stroke unit with age ≥18 years, clinical or neuroimaging proof of stroke with onset in the last 24 h (determined by a neurology consultant)	Individuals admitted to the neurological ward, age ≥18 years, and less than two cardiovascular risk factors (i.e. hypertension, atrial fibrillation, hypercholesterolemia, diabetes mellitus, smoking)	All: antibiotic therapy in the previous 30 days, chemotherapy, probiotic treatment, severe anemia and known trimethylaminuria. Controls: ischemic stroke or myocardial infarction in their medical history.
Xiang et al. 2020	China	10	16	73 ± 20.74	71 ± 20.0	5 (50%)	7 (43.8%)	25 ± 4.67	23.9 ± 2.22	Stroke patients confirmed by magnetic resonance imaging or brain computed tomography.	NR	All: respiratory or renal failure, recent cardiac event, immune system condition, intestinal disease or severe liver dysfunction, probiotics or antibiotics within 1 month prior to admission.
Haak el tal. 2021	Netherlands	349	51	71.33 ± 13.33	71 ± 5.93	194 (55.6%)	29 (56.9%)	NR	NR	Patients aged 18 years or older, with clinical symptoms of a stroke, an onset of symptoms less than 24 h, and a score of 1 or more on the National Institutes of Health Stroke Scale.	Healthy non-hospitalized age- and sex-matched controls with similar cardiovascular risk profiles but without active signs of stroke	Patients: clinical signs of infection on hospital admission requiring antibiotic therapy, use of antimicrobials less than 24 h before admission, pregnancy, hypersensitivity for cephalosporins, previous anaphylaxis for penicillin derivatives, subarachnoid hemorrhage, and imminent death.
Huang, Y. et al. 2021	China	27	19	NR	NR	NR	NR	NR	NR	Patients enrolled from the first affiliated hospital of Shantou University, with 40–90 years old, ischemic ST, and infarcts in non-strategic brain regions.	Healthy individuals enrolled from the first affiliated hospital of Shantou University	Patients: preexisting dementia history and infarct of strategic regions; antibiotics or probiotics within 3 months; restrictive diet, gastrointestinal surgery, recent infection, psychosis, severe life-threatening illnesses, communication deficits, and pregnancy.
Sun et al. 2021	China	953	953	63.14 ± 9.26	63.32 ± 8.47	544 (57.08%)	544 (57.08%)	23.68 ± 2.98	23.84 ± 2.86	Patients with first acute ischemic stroke admitted to People's Hospital of Shenzhen	Individuals recruited from the general population who attended an annual health examination at	All: myocardial infarction, heart failure, malignant tumor, other systemic diseases, or who are using

											the hospital physical examination center.	antibiotics within 3 months
Tan et al. 2021	China	140	92	59 ± 20	60 ± 13	95 (67.9%)	51 (55.4%)	NR	NR	Patients aged >18 years and admitted within 48 hours of stroke onset.	Healthy individuals	Patients: antibiotics and prebiotics in the past 3months, gastrointestinal symptoms in the past 3 months, neurological diseases, gut diseases, advanced cancer, failed to stool collection. Controls: prior history of myocardial infarction, stroke, or gut diseases; antibiotics/ prebiotics/ probiotics taken, or gastrointestinal symptoms experienced in the past 3 months
Xu et al. 2021	China	85	59	NR	NR	40 (65.5%)	19 (37.3%)	NR	NR	Patients who experienced acute cerebral ischemia within 7 days from symptom onset	Non-stroke participants with or without hypertension and/or diabetes, but without atherogenesis of carotid arteries, atrial fibrillation, and present or history of coronary or other vascular diseases	Patients: infectious diseases, cancer, congestive cardiac failure, respiratory failure, renal failure, severe liver dysfunction, or use of probiotics or antibiotics within 2 months before admission.
Zhang, J. et al. 2021	China	351	150	65 ± 22.96	65 ± 22.96	177 (50.4%)	75 (50.0%)	25.57 ± 2.74	24.5 ± 2.74	Patients with first-ever ischemic stroke (symptoms onset was less than 24 h) included at Xiamen Fifth Hospital	Age and gender-matched healthy volunteers from Xiamen Fifth Hospital	Patients: malignant tumor; metabolic syndrome; renal and liver insufficiency; any surgical procedure within the previous month; inability to consent, other neurological diseases, presence of cardiogenic shock, sepsis, pneumonia; acute coronary syndromes
Chen et al. 2022	China	31	31	56.87 ± 10.25	52.65 ± 9.92	23 (74.19%)	22 (70.97%)	NR	NR	Patients diagnosed with acute cerebral hemorrhage confirmed by head computed tomography and/or magnetic resonance imaging	Healthy volunteers matching the sex and age of patients with intracerebral hemorrhage	All: antibiotics or probiotics within 1 month before the collection of samples, more than 3 weeks after the onset of the disease, multiple organ failure or death within 3 weeks after the disease, traumatic cerebral hemorrhage, cerebral amyloid angiopathy, secondary cerebral

												hemorrhage, neurodegenerative diseases, mental diseases, hepatorenal failure, autoimmune diseases, malignant tumors, thyroid diseases and pregnancy. Controls: previous history of cerebrovascular diseases, acute and chronic gastrointestinal diseases and previous gastrointestinal resection.
Luo, J. et al. 2022	China	64	23	59.88 ± 10.66	NR	38 (59.37%)	6 (26.09%)	NR	NR	Intracerebral hemorrhage was diagnosed according to American Heart Association/American Stroke Association guidelines. The inclusion criteria were as follows (1) age >18 years, (2) admission within 7 days of ICH onset, and (3) informed consent obtained, and the retention of biological samples completed.	NR	Patients: intracerebral hemorrhage caused by brain tumor, brain trauma, blood diseases, cerebrovascular malformation, or aneurysm; any antibiotics, prebiotics, or probiotics treatment within four weeks before admission; active infection within two weeks before admission; liver and kidney dysfunction; history of gastrointestinal diseases in the last 3 months; history of immune-related diseases or receiving immunotherapy.
Zhao et al. 2022	China	30	30	60.67 ± 9.63	62.0 ± 10.37	21 (70%)	18 (60%)	NR	NR	Patients diagnosed by skull computed tomography examination with not suffer from any pre-existing metabolic or gut disease.	Healthy volunteers examined to ensure no metabolic, cardiovascular, or cerebrovascular diseases or cancer	All: antibiotics or probiotics at least one month prior to the collection of biospecimens.
Zheng, Q. et al. 2022	China	33	30	40.93 ± 8.57	41.33 ± 8.07	17 (51.5%)	20 (66.7%)	24.55 ± 2.02	24.34 ± 2.31	Patients diagnosed and treated in the Department of Neurology	Age, sex, and stroke risk factor frequency-matched subjects.	Patients: stroke with determined cause, lacunar brain infarction, history of ischemic stroke or transient ischemic attack, other neurological diseases, infection within 3 months, antibiotics or probiotics, history of GI operation, history of

												inflammatory bowel disease, and pregnancy

Table S5. Metabolic biomarkers of the studies included in the systematic review and meta-analysis.

	Sample size, n		Alanine Transaminase (ALT); U/L		Aspartate Transaminase (AST), U/L		Total cholesterol (TC), mmol/L		Triglycerides (TG), mmol/L		High-density lipid cholesterol (HDL), mmol/L		Low-density lipid cholesterol (LDL), mmol/L		Creatinine, umol/L		Fasting glucose (FG), mmol/L	
	CVD	HC	CVD	HC	CVD	HC	CVD	HC	CVD	HC	CVD	HC	CVD	HC	CVD	HC	CVD	HC
			Mean ± SD	Mean ± SD	Mean ± SD	Mean ± SD	Mean ± SD	Mean ± SD	Mean ± SD	Mean ± SD	Mean ± SD	Mean ± SD	Mean ± SD	Mean ± SD	Mean ± SD	Mean ± SD	Mean ± SD	Mean ± SD
**Acute Coronary Syndrome**																		
Dai et al., 2020	254	247	NR	NR	NR	NR	NR	NR	NR	NR	NR	NR	NR	NR	NR	NR	NR	NR
Gao et al., 2020	60	30	32.26±26.4	15.6±10.74	NR	NR	4.51±1.23	4.71±0.95	1.69±0.87	1.44±0.76	1.02±0.29	1.33±0.35	2.92±1.09	3.09±0.79	80.47±23.52	72.07±17.77	6.07±1.73	5.04±0.36
Han et al., 2021	30	30	NR	NR	NR	NR	4.6±1.33	5±0.9	1.8±0.76	1.9±1.66	1±0.59	43±58.11	2.6±0.87	2.9±0.8	76.1±35.38	67.4±18.35	6.6±2.21	5.2±1.3
Chiu et al., 2022	19	25	NR	NR	NR	NR	NR	NR	1.43±0.63	1.69±1.26	1.21±0.32	1.21±0.33	2.46±1.01	2.43±0.75	122.9±2.08	84.9±0.27	NR	NR
Liu, C et al., 2022	117	78	40.67±21.48	21.33±9.63	93±75.56	19.33±4.89	4.37±0.89	3.87±1.4	1.33±0.67	1.83±1.11	1.1±0.3	1.06±0.3	2.57±0.74	1.97±1.04	84±18.52	78.83±16	6.87±1.63	5.93±1.48
Qian et al., 2022	20	20	143.5±605.26	21.1±32.47	30.04±35.11	18.55±48.48	NR	NR	1.4±2.28	1.48±1.39	1.3±1.43	1.5±4.16	2.5±3.76	1.9±3.58	NR	NR	NR	NR
Tang et al., 2022	51	55	22±2.96	19±8.88	NR	NR	NR	NR	NR	NR	NR	NR	NR	NR	76.67±22.96	70.33±13.33	8.84±2.43	5.4±0.44
**Atherosclerosis**																		
Karlsson et al., 2012	12	13	NR	NR	NR	NR	4.62±1.59	5.59±1.2	1.72±1.08	1.19±0.74	1.32±0.26	1.67±0.44	2.53±1.44	3.39±1.05	NR	NR	NR	NR
Ji, L et al., 2021	32	32	NR	NR	NR	NR	3.2±0.7	4.5±1.3	1.2±0.6	1.3±0.8	1±0.2	1.3±0.3	1.8±0.6	2.9±0.6	NR	NR	NR	NR
Stø et al., 2021	43	38	NR	NR	NR	NR	4.1±1	5.2±0.88	NR	NR	NR	NR	2.2±0.85	3.1±0.85	NR	NR	NR	NR
**Atrial Fibrillation**																		
Zuo, K et al., 2019	50	50	NR	NR	NR	NR	4.13±1.05	4.82±0.96	1.4±0.64	1.21±0.76	NR	NR	2.32±1	2.37±0.67	69.44±13.98	73.17±21.85	5.09±0.99	5.08±0.73
Zuo, K et al., 2020	20	50	20.42±11.67	18.67±9.63	NR	NR	4.06±0.76	4.81±0.96	NR	NR	NR	NR	2.36±0.84	2.37±0.67	71.47±9.37	73.17±21.85	5.1±1.03	5.08±0.73
Huang et al., 2022	36	30	17.58±8.7	17.5±5.56	NR	NR	4.08±0.95	4.92±0.96	1.35±0.49	1.98±1.42	NR	NR	2.61±0.77	3.1±0.75	82.67±19.63	76.17±13.7	NR	NR
**Coronary Artery Disease**																		
Yoshida et al., 2018	30	30	27.8±13.7	22.9±5.6	27.3±17.1	23.2±11.6	4.19±0.72	4.74±0.9	1.7±0.86	1.64±0.83	1.31±0.5	1.36±0.36	2.38±0.68	2.94±0.92	76.93±14.15	83.11±22.99	NR	NR
Zhang et al., 2019	24	23	20.45±13.28	18.09±10.23	20.45±12.73	21.13±10	NR	NR	1.4±0.79	1.12±0.52	1.03±0.24	1.09±0.26	2.37±0.72	2.55±0.88	82.79±31.71	70.26±16.94	NR	NR
Liu, F et al., 2020	18	23	NR	NR	NR	NR	4.17±1.12	4.54±0.81	1.5±0.92	1.27±0.58	1.03±0.3	1.18±0.2	2.3±1.11	3.46±0.68	46.04±9.1	75.27±17.29	5.49±0.97	4.76±0.6
Zheng et al., 2020	152	105	NR	NR	NR	NR	4.14±0.97	3.51±0.98	1.68±1.04	1.66±0.96	1.05±0.28	1.17±0.29	2.65±0.83	2.14±0.84	67.36±17.61	62.35±14.11	5.74±2.36	5.02±0.85
Nguyen et al., 2021	45	35	NR	NR	NR	NR	5.2±0.44	5.2±0.44	1.5±0.26	1.4±0.12	1.6±0.13	1.6±0.13	2.9±0.26	2.3±0.23	81±3.2	85±10	NR	NR
Sawicka-Smiarowska	169	166	24.53±10.37	22.53±9.41	23.23±6.74	22.87±5.56	3.99±0.95	5.07±1.17	1.25±0.7	1.3±0.56	1.3±0.39	1.5±0.4	2.27±0.76	3.39±1.17	85.67±16.89	74.8±13.93	5.93±0.88	5.7±0.59

et al., 2021																	
Tian et al., 2021	71	55	NR	NR	NR	NR	3.96±1.18	4.61±0.97	3.87±0.69	1.31±0.57	1.01±0.27	1.14±0.25	2.26±0.74	2.69±0.7	NR	NR	6±1.26	6.55±1.81
Cheng et al., 2022	14	18	49.58±10.07	55.22±12.36	40.89±44.27	24.69±3.61	4.89±0.28	4.68±0.19	1.68±0.26	1.43±0.14	1.08±0.07	1.31±0.04	3.35±0.29	3.46±0.21	NR	NR	NR	NR
Li, W et al., 2022	19	19	23.46±9	18±6.5	24±7.84	21±3	NR	NR	1.38±1.25	0.94±0.35	1.28±0.25	1.35±0.26	3.09±0.71	2.64±0.51	60±13	64±12.5	5.57±0.71	5.27±0.45
Liu, L et al., 2022	10	10	27.71±12.81	28.44±26.09	NR	NR	5.07±1.17	5.67±1.11	1.09±0.51	2.74±3.45	1.11±0.16	1.26±0.32	2.83±1.04	3.36±1.45	81.45±28.08	76.38±13.95	6.02±1.61	5.76±1.88
Tian et al., 2022	51	14	NR	NR	NR	NR	3.72±0.86	4.76±0.9	1.44±0.61	1.34±0.9	0.99±0.2	1.04±0.19	1.98±0.49	2.74±0.69	70.33±12.59	69.5±20.37	NR	NR
Yu et al., 2022	36	9	29.04±12.89	22.85±11.77	33.89±22.95	24.5±6.04	4.9±1.18	4.85±1.14	2.07±0.88	0.9±0.3	1.08±0.19	1.34±0.26	1.08±0.067	1.32±0.22	68.48±14.31	65.83±16.12	NR	NR
Zhong et al., 2022	42	46	NR	NR	NR	NR	4.55±0.47	4.96±0.44	1.52±0.33	1.21±0.3	1.32±0.13	1.43±0.16	2.63±0.39	2.94±0.4	NR	NR	6.87±0.94	5.71±0.62
**Heart Failure**																	
Troseid et al., 2015	155	33	NR	NR	NR	NR	NR	NR	NR	NR	NR	NR	NR	NR	NR	NR	NR	NR
Cui et al., 2018	53	41	22.67±13.33	19±10.37	NR	NR	3.66±0.81	5.39±1.13	1.23±0.62	1.22±0.61	0.93±0.31	1.25±0.25	2.27±0.88	2.66±0.49	139.69±23.34	75.25±19.44	5.92±1.75	5.2±0.57
Katsimichas et al., 2018	28	19	NR	NR	NR	NR	NR	NR	NR	NR	NR	NR	NR	NR	NR	NR	NR	NR
Hayashi et al., 2018	22	11	NR	NR	NR	NR	4.44±0.89	4.97±0.8	1.05±0.36	1.78±0.8	1.35±0.42	1.38±0.26	4.44±0.69	3.05±0.81	106.1±35.37	88.42±26.53	NR	NR
Dong et al., 2020	61	57	22.27±12.65	19.06±17.09	NR	NR	4.46±1.31	4.93±1	1.74±1.12	1.85±1.17	NR	NR	NR	NR	79.49±46.71	61.22±11.57	NR	NR
Emoto et al., 2021	22	11	NR	NR	NR	NR	NR	NR	NR	NR	NR	NR	NR	NR	270±37.99	272.19±436.88	NR	NR
Wang et al., 2021	25	25	NR	NR	NR	NR	NR	NR	NR	NR	NR	NR	NR	NR	NR	NR	NR	NR
**Hypertension**																	
Li, J et al., 2017	99	41	NR	NR	NR	NR	5.6±1.8	5.4±0.95	2.1±2.1	1.7±2.1	1.38±0.4	1.25±0.25	3.8±9.8	2.7±0.79	NR	NR	5.8±1.4	5.3±0.86
Yan et al., 2017	60	60	NR	NR	NR	NR	5.02±0.97	5.07±0.97	1.87±0.85	1.52±0.69	1.15±0.24	1.23±0.29	3.08±0.76	3.04±0.71	NR	NR	6.37±2.39	6.19±2.09
Zuo, K et al., 2019	34	15	21.03±9.26	19.92±11.11	NR	NR	5.19±1.04	5.38±0.75	NR	NR	NR	NR	NR	NR	69.03±20.3	67.72±10.56	5.75±0.89	5.21±0.93
Calderon-Perez et al., 2020	29	32	NR	NR	NR	NR	5.16±1.14	4.7±0.9	1.1±0.44	0.91±0.48	1.62±0.36	1.68±0.47	3.2±0.55	2.6±0.86	NR	NR	5.6±11.3	4.5±7.5
**Stroke**																	
Nie et al, 2018	622	622	NR	NR	NR	NR	5.8±1.2	5.6±1.2	1.6±0.9	1.6±1	1.3±0.4	1.4±0.4	NR	NR	NR	NR	6.3±2.5	5.9±1.7
Li, N et al., 2019	30	30	NR	NR	NR	NR	NR	NR	1.24±0.47	1.33±0.31	1.1±0.19	1.29±0.24	2.2±0.68	2.3±0.5	NR	NR	4.92±0.67	5.2±0.76
Schneider et al., 2020	193	100	NR	NR	NR	NR	NR	NR	NR	NR	NR	NR	NR	NR	NR	NR	NR	NR
Sun et al., 2021	953	953	NR	NR	NR	NR	4.95±1.13	5.35±1.13	1.42±0.64	1.34±0.72	1.12±0.25	1.39±0.34	2.92±0.88	3.02±0.96	NR	NR	6.43±2.54	5.92±1.43
Tan et al., 2021	140	92	NR	NR	NR	NR	NR	NR	NR	NR	NR	NR	NR	NR	NR	NR	NR	NR

Xu et al., 2021	61	51	NR	NR	NR	NR	4.64±0.13	4.52±0.12	NR	NR	1.05±0.032	1.19±0.04	2.89±0.12	2.67±0.78	62.5±NR	60±NR	NR	NR
Zhang et al., 2021	351	150	NR	NR	NR	NR	NR	NR	NR	NR	NR	NR	NR	NR	NR	NR	6.1±1.66	5.67±1.06
Luo et al., 2022	64	23	NR	NR	NR	NR	4.45±1.55	NR	0.99±1.14	NR	1.25±0.46	NR	2.59±0.89	NR	NR	NR	NR	NR
Zhao et al., 2022	30	30	NR	NR	NR	NR	NR	NR	1.24±0.39	1.34±0.38	1.11±0.68	1.31±0.24	2.2±0.68	2.31±0.5	NR	NR	4.82±0.73	5.15±0.58
Zheng et al., 2022	30	33	NR	NR	NR	NR	NR	NR	NR	NR	NR	NR	NR	NR	NR	NR	NR	NR

Table S6. Information summary of all included studies that performed metagenomic analysis.

Abbreviations: ASV, Amplicon Sequence Variant; CAZy, Carbohydrate-Active Enzyme; COGs, Clusters of Orthologous Genes; EggNOG, Evolutionary genealogy of genes: Non-supervised Orthologous Groups; GOLD, Genomes Online Database; HMP, Human Microbiome Project; KEGG, Kyoto Encyclopedia of Genes and Genomes; NCBI, National Center for Biotechnology Information; NR, Not reported; OTU, Operational taxonomic unit; RNA, Ribonucleic acid; TIGRFAM, The Institute for Genomic Research Protein Family; VFDB, Virulence Factor Database.

Article	DNA extraction kit	Sequencing type	Sequencing region	Bioinformatic databases	Clustering method
**Acute Coronary Syndrome**					
Alhmoud et al., 2019	Zymo Research Fungal/Bacterial Miniprep Kit	16S rRNA gene sequencing	NR	NR	NR
Gao et al., 2020	Cetyltrimethyl Ammonium Bromide	Shotgun metagenomic sequencing	Whole genome	GOLD, SILVA	OTU (97%)
Kwun, J. et al., 2020	QIAGEN DNeasy PowerSoil Kit	16S rRNA gene sequencing	V3-V4	NR	OTU
Han, Y. et al., 2021	NR	16S rRNA gene sequencing	V3-V4	SILVA, COGs	OTU (97%)
Chiu et al., 2022	E.Z.N.A.® Stool DNA Kit	16S rRNA gene sequencing	V3-V4	NR	OTU (97%)
Liu, C. et al., 2022	QIAGEN QIAamp Fast DNA Stool Mini Kit	16S rRNA gene sequencing; Shotgun metagenomic sequencing	V3-V4 Whole genome	MetaCyc, VFDB	ASV
Qian et al., 2022	NR	16S rRNA gene sequencing	V4	NR	OTU (97%)
**Atherosclerosis**					
Karlsson et al., 2012	NR	Shotgun metagenomic sequencing	Whole genome	NCBI, HMP, CAZy, KEGG	NR
Jie et al., 2017	QIAGEN QIAamp DNA Stool Mini Kit	Shotgun metagenomic sequencing	Whole genome	UniProt, NCBI, VFDB, KEGG	NR
Ji, L. et al., 2021	QIAGEN QIAamp Fast DNA Stool Mini Kit	16S rRNA sequencing	V3-V4	NR	OTU
Stø et al., 2021	ZymoBIOMICS DNA Miniprep Kit	16S rRNA sequencing	V3-V4	NR	ASV
**Atrial Fibrillation**					
Zuo, K. et al., 2019	TIANGEN TIANamp Stool DNA Kit	Shotgun metagenomic sequencing	Whole genome	KEGG, EggNOG, NCBI	NR
Zuo, K. et al., 2020	TIANGEN TIANamp Stool DNA Kit	Shotgun metagenomic sequencing	Whole genome	KEGG	NR
Tabata et al., 2021	Bead beating method	16S rRNA gene sequencing	V3-V4	SILVA	OTU (97%)
Huang, K. et al., 2022	TIANGEN TIANamp Stool DNA Kit	16S rRNA gene sequencing	V3-V4	SILVA	OTU (97%)
**Coronary Artery Disease**					
Yoshida et al., 2018	NR	16S rRNA gene sequencing	V3-V4	SILVA, Greengenes, KEGG	OTU (97%)
Zhu, Q. et al., 2018	QIAGEN QIAamp DNA Stool Mini Kit	16S rRNA gene sequencing	V4	NR	OTU (97%)
Zhang, Y. et al., 2019	PSP Spin Stool DNA Plus Kit	16S rRNA gene sequencing	NR	NR	OTU (97%)
Liu, F. et al., 2020	QIAGEN QIAamp DNA Stool Mini Kit	16S rRNA gene sequencing	V3-V4	Greengenes, Unigenes, KEGG	OTU (97%)
Zheng et al., 2020	Bead beating method	16S rRNA gene sequencing	V3-V4	NR	OTU
Choroszy et al., 2021	QIAGEN QIAamp DNA Stool Mini Kit	16S rRNA gene sequencing	V3-V4	SILVA	NR
Nguyen, C. et al., 2021	NR	16S rRNA gene sequencing	V3-V4	NR	NR
Sawicka-Smiarowska et al., 2021	PSP Spin Stool DNA Plus Kit	16S rRNA gene sequencing	V3-V4	SILVA	OTU
Tian et al., 2021	QIAGEN QIAamp DNA Stool Mini Kit	Shotgun metagenomic sequencing	Whole genome	NR	NR
Cheng et al., 2022	QIAGEN QIAamp DNA Stool Mini Kit	Shotgun metagenomic sequencing	Whole genome	NR	NR
Li, W. et al., 2022	Cetyltrimethylammonium Bromide/Sodium Dodecyl Sulfate	Shotgun metagenomic sequencing	Whole genome	NCBI, KEGG, CAZy	NR

Liu, L. et al., 2022	QIAGEN QIAamp DNA Stool Mini Kit	16S rRNA gene sequencing	V3-V4	SILVA	OTU (97%)
Liu, X. et al., 2022	QIAGEN QIAamp DNA Stool Mini Kit	Shotgun metagenomic sequencing	Whole genome	NR	NR
Tian et al., 2022	NR	16S rRNA gene sequencing	V3-V4	NR	OTU (97%)
Yu et al., 2022	NR	16S rRNA gene sequencing	V3-V4	SILVA, KEGG	OTU (97%)
Zhong et al., 2022	Fecal Genomic DNA Extraction Kit	Shotgun metagenomic sequencing	Whole genome	KEGG	NR
**Heart Failure**					
Cui et al., 2018	TIANGEN TIANamp Stool DNA Kit	Shotgun metagenomic sequencing	Whole genome	NCBI, KEGG	N/R
Hayashi et al., 2018	NR	16S rRNA gene sequencing	NR	SILVA, Greengenes, KEGG, COGs	OTU (97%)
Katsimichas et al., 2018	QIAGEN DNeasy PowerSoil Kit	16S rRNA gene sequencing	V1-V2	Greengenes	OTU (97%)
Emoto et al., 2021	NR	Shotgun metagenomic sequencing	Whole genome	NR	NR
Wang et al., 2021	QIAGEN QIAamp Fast DNA Stool Mini Kit	16S rRNA gene sequencing	V3-V4	SILVA	OTU (97%)
Sun et al., 2022	TIANGEN TIANamp Stool DNA Kit	16S rRNA gene sequencing	V3-V4	SILVA	OTU (97%)
Peng et al., 2023	Quant-iT PicoGreen dsDNA Assay Kits	16S rRNA gene sequencing	V3-V4	SILVA	OTU (97%)
**Hypertension**					
Li, J. et al., 2017	TIANGEN TIANamp Stool DNA Kit	Shotgun metagenomic sequencing	Whole genome	NCBI, KEGG, CAZy, Greengenes	N/R
Yan et al., 2017	QIAGEN QIAamp DNA Mini Kit	Shotgun metagenomic sequencing	Whole genome	NCBI, KEGG	N/R
Zuo, K. et al., 2019	NR	Shotgun metagenomic sequencing	Whole genome	NR	N/R
Calderon-Perez et al., 2020	MagNA Pure LC DNA Isolation Kit III	16S rRNA gene sequencing	V3-V4	SILVA, TIGRFAMs	ASV
Takagi et al., 2020	NucleoSpin Microbial DNA KIT	16S rRNA gene sequencing	V3-V4	Greengenes, KEGG	OTU
Nakai et al., 2021	QIAGEN DNeasy PowerSoil Kit	16S rRNA gene sequencing	V4-V5	SILVA	OTU (99%)
**Stroke**					
Yin et al., 2015	QIAGEN DNeasy PowerSoil Kit	16S rRNA gene sequencing	V4	NCBI, SILVA	OTU
Ji et al., 2017	TIANGEN TIANamp Stool DNA Kit	16S rRNA gene sequencing	V4	NR	OTU
Wang et al., 2018	TIANGEN TIANamp Stool DNA Kit	16S/18S/ITS rRNA gene sequencing	V3-V5/V9/ITS1-2	NR	NR
Li, N. et al., 2019	Cathepsin B Activity Assay Kit	16S rRNA gene sequencing	V1-V2	SILVA	OTU (97%)
Li, H. et al., 2020	QIAGEN QIAamp Fast DNA Stool Mini Kit	16S rRNA gene sequencing	NR	NR	OTU
Xiang et al., 2020	QIAGEN QIAamp Fast DNA Stool Mini Kit	16S rRNA gene sequencing	V3-V4	NR	OTU (97%)
Haak et al., 2021	Bead beating method	16S rRNA gene sequencing	V3-V4	SILVA	ASVs
Huang, Y. et al., 2021	NR	16S rRNA gene sequencing	V3-V4	NR	OTU
Tan et al., 2021	QIAGEN DNeasy PowerSoil Kit	16S rRNA gene sequencing	V4	NR	NR
Xu et al., 2021	QIAGEN QIAamp Fast DNA Stool Mini Kit	16S rRNA gene sequencing	V3-V4	SILVA	OTU (97%)
Chen et al., 2022	E-Z 96 Mag-Bind Soil DNA Kit	16S rRNA gene sequencing	V3-V4	Greengenes, NCBI, KEGG, CAZy	ASV
Luo, J. et al., 2022	TIANGEN Magnetic Soil and Stool DNA Kit	16S rRNA gene sequencing	V3-V4	NR	NR
Zhao et al., 2022	GNOME DNA Isolation Kit	Shotgun metagenomic sequencing	Whole genome	NCBI	NR
Zheng, Q. et al., 2022	NR	16S rRNA gene sequencing	V3-V4	NR	OTU (97%)

Table S7. Information summary of all included studies that performed metabolomic analysis.

Abbreviations: CE, Capillary electrophoresis; EID, Electron induced dissociation; ESI, Electrospray ionisation; FID, Flame ionisation detector; LC, Liquid chromatography; GC, Gas chromatography; HPLC, High-performance liquid chromatography; MS, Mass spectrometry; NMR, Nuclear magnetic resonance; NR, Not reported; QTOF, Quadrupole Time-of-Flight; SCFA, Short-chain fatty acid; TMA, Trimethylamine; TML, Trimethyl lysine; TMAO, Trimethylamine N-oxide; TMAVA, N, N, N-trimethyl-5-aminovaleric acid; UHPLC, Ultra-High-Performance Liquid Chromatography.

Article	Sample type	Measurements	Analytical technique
**Acute Coronary Syndrome**			
Alhmoud et al., 2019	Serum	TMAO	LC/MS
Dai et al., 2020	Plasma	TMA, TMAO, Choline, Betaine	UHPLC
Gao et al., 2020	Serum	TMAO	HPLC/MS
Liu, C. et al., 2022	Plasma, Stool	TMA, TMAO, Choline, Betaine, Carnitine; SCFA	GC/EID
Qian et al., 2022	Plasma	TMAO, SCFA	LC/MS/MS
Tang et al., 2022	Serum	TMAO	LC/MS/MS
**Atherosclerosis**			
Ji, L. et al., 2021	Plasma	Untargeted	UHPLC/QTOF/MS
Stø et al., 2021	Plasma, Stool	SCFA	UPLC/MS/MS, GC/MS
**Atrial Fibrillation**			
Zuo, K. et al., 2019	Serum, Stool	Chenodeoxycholic acid, Lysophosphatidylcholine, Cholic acid, Oleic acid, Linoleic acid, and α-Linolenic acid	LC/MS
Buttner et al., 2020	Plasma	TMAO	ESI/MS/MS
Zuo, K. et al., 2020	Serum, Stool	Chenodeoxycholic acid, α-Linolenic acid, Myoinositol, Vitamin D3, Choline	LC/MS
Huang, K. et al., 2022	Stool	Flavin adenine dinucleotide, Riboflavin-5-phosphate, Inosine, Dehydroepiandrosterone, Estradiol, Caffeine, Salicylic acid, Ascorbic acid, Eicosapentamoic acid, Oleanolic acid, N-Acetylmethionine, 3-Hydroxy-3-methylbutanoic acid	LC/MS
**Coronary Artery Disease**			
Yoshida et al., 2018	Stool	SCFA	LC/MS
Liu, F. et al., 2020	Plasma	TMAO, Choline, Betaine, Creatinine, Carnitine	LC/MS/MS
Nguyen, C. et al., 2021	Serum, Stool	Bile acids	HPLC MS/MS
Sawicka-Smiarowska et al., 2021	Serum	Untargeted	LC/MS/MS
Tian et al., 2021	Serum	Untargeted	LC/MS
Cheng et al., 2022	Plasma	TMAO, Betaine, Choline, Carnitine	NR
Liu, L. et al., 2022	Serum, Stool	SCFA	LC/MS/MS
Liu, X. et al., 2022	Plasma, Stool	Untargeted	UPLC/MS
Tian et al., 2022	Serum	TMAO, Choline, TMAVA, Butyrobetaine, TML, DMG, Carnitine, Betaine	LC/MS/MS
Zhong et al., 2022	Serum	Untargeted	UPLC/MS/MS
**Heart Failure**			
Trøseid et al., 2015	Plasma	TMAO, Choline, Betaine	LC/MS/MS
Cui et al., 2018	Plasma, Stool	Para-Tolyl octanoate, Niacin, Cinnamic acid, Orotic acid, Sphingosine 1−phosphate, Ricinoleic acid	LC/MS
Hayashi et al., 2018	Plasma	TMAO, Choline, Carnitine, Tryptophan, Indoxyl sulfate	CE-TOF/MS
Dong et al., 2020	Plasma	TMAO	LC/MS/MS
Emoto et al., 2021	Plasma	TMAO	NR
Wang et al., 2021	Serum	Untargeted	LC/MS/MS
Peng et al., 2023	Stool	SCFA	GC/MS

**Hypertension**			
Li, J. et al., 2017	Serum	Phosphatidylserine, 3,4,5-Trimethoxycinnamic acid, Lysophosphatidylcholine, S-Carboxymethyl-L-cysteine, Lysophosphatidylethanolamine, N-α-acetyl-L-arginine, Stearic acid, Phosphatidic acid, Glucoside	LC/MS
Zuo, K. et al., 2019	Stool	Vitamin D3	LC/MS
Calderon-Perez et al., 2020	Plasma, Stool	SCFA, TMAO	GC-FID, UPLC-MS/MS
Nakai et al., 2021	Plasma, Stool	SCFA	GC-FID
**Stroke**			
Yin et al., 2015	Plasma	TMAO	LC/MS
Nie et al., 2018	Serum	TMAO, Choline, Carnitine	LC/MS/MS
Schneider et al., 2020	Plasma	TMAO	LC/MS/MS
Haak et al., 2021	Plasma	TMAO	NMR, LC/MS
Sun et al., 2021	Plasma	TMAO	LC/MS/MS
Tan et al., 2021	Stool	SCFA	GC/MS
Xu et al., 2021	Plasma	TMAO	LC/MS/MS
Zhang et al., 2021	Plasma	TMAO, Choline, Betaine	LC/MS/MS
Chen et al., 2022	Serum	Untargeted	LC/MS/MS
Zhao et al., 2022	Plasma, Urine, Stool	Phenylacetic acid	GC/MS

Table S8. Quality assessment of observational cohort and cross-sectional studies included in the systematic review and meta-analysis.

	Trøseid et al. 2015	Li, J. et al. 2017	Wang et al. 2018	Zuo, K., Li, J. et al., 2019	Calderon-Perez et al. 2020	Dong et al. 2020	Gao et al., 2020	Takagi et al. 2020	Zuo, K. Yin, X. et al. 2020	Emoto et al. 2021	Nguyen et al. 2021	Tan et al. 2021	Zhang, J. et al. 2021	Cheng et al. 2022	Huang, K. et al. 2022	Sun et al. 2022	Peng et al. 2023
**1**	yes	yes	yes	yes	yes	yes	yes	yes	yes	yes	yes	yes	yes	yes	yes	yes	yes
**2**	yes	yes	yes	yes	yes	yes	yes	yes	yes	yes	yes	yes	yes	yes	yes	yes	yes
**3**	yes	yes	no	yes	yes	yes	yes	yes	yes	no	yes	yes	yes	no	yes	yes	yes
**4**	yes	yes	yes	yes	yes	yes	yes	yes	yes	yes	yes	yes	yes	yes	yes	yes	yes
**5**	no	no	no	no	no	no	yes	no	no	no	no	no	no	no	no	no	no
**6**	NA	NA	NA	NA	NA	NA	NA	NA	NA	NA	NA	NA	NA	NA	NA	NA	NA
**7**	no	no	no	no	no	no	no	no	no	no	no	no	no	no	no	no	no
**8**	yes	yes	no	yes	no	no	yes	yes	yes	yes	no	no	no	yes	no	no	yes
**9**	yes	yes	yes	yes	yes	yes	yes	yes	yes	yes	yes	yes	yes	yes	yes	yes	yes
**10**	no	no	no	no	no	no	no	yes	yes	no	no	no	no	no	no	no	no
**11**	yes	yes	yes	no	no	yes	yes	no	no	yes	no	no	no	yes	yes	yes	yes
**12**	NA	NA	NA	NA	NA	NA	NA	NA	NA	NA	NA	NA	NA	NA	NA	NA	NA
**13**	NA	NA	NA	NA	NA	NA	NA	NA	NA	NA	NA	NA	NA	NA	NA	NA	NA
**14**	yes	yes	no	yes	yes	yes	yes	no	yes	no	yes	yes	yes	no	no	no	no
**Quality rating**	Good	Good	Poor	Fair	Fair	Fair	Good	Fair	Good	Fair	Fair	Fair	Fair	Fair	Fair	Fair	Good

Table S9. Quality assessment of control-case studies included in the systematic review and meta-analysis.

(A) Subset of the first 25 studies, ordered by year and alphabet. (B) The subset of the remaining 25 studies was also ordered similarly.

A

	**Karlsson et al., 2012**	**Yin et al., 2015**	**Ji et al., 2017**	**Jie et al., 2017**	**Yan et al., 2017**	**Cui et al., 2018**	**Hayashi et al., 2018**	**Karsimichas et al., 2018**	**Yoshida et al., 2018**	**Zhu, Q., et al., 2018**	**Nie et al., 2018**	**Alhmoud et al., 2019**	**Zuo, K. et al., 2019**	**Li, N. et al., 2019**	**Zhang, Y. et al., 2019**	**Buttner et al., 2020**	**Dai et al., 2020**	**Kwun et al., 2020**	**Li, H. et al. 2020**	**Liu, F. et al. 2020**	**Schneider et al., 2020**	**Xiang et al 2020**	**Zhang et al., 2020**	**Haak et al., 2021**	**Han et al., 2021**
**1**	yes	yes	yes	yes	yes	yes	yes	yes	yes	yes	yes	yes	yes	yes	yes	yes	yes	yes	yes	yes	yes	yes	yes	yes	yes
**2**	yes	yes	yes	yes	yes	yes	yes	yes	yes	yes	yes	yes	yes	yes	yes	yes	yes	yes	yes	yes	yes	yes	yes	yes	yes
**3**	no	no	no	no	no	no	no	no	no	no	no	yes	no	no	no	yes	no	no	no	no	no	no	no	no	no
**4**	yes	yes	yes	yes	yes	yes	yes	yes	yes	yes	yes	yes	yes	yes	yes	yes	yes	yes	yes	yes	yes	yes	yes	yes	yes
**5**	yes	yes	yes	yes	yes	yes	yes	yes	yes	yes	yes	yes	yes	yes	yes	yes	yes	yes	yes	yes	yes	yes	yes	yes	yes
**6**	yes	yes	yes	yes	yes	yes	yes	yes	yes	yes	yes	yes	yes	yes	yes	yes	yes	yes	yes	yes	yes	yes	yes	yes	yes
**7**	no	no	no	no	no	no	no	no	no	no	no	no	no	no	no	no	no	no	no	no	no	no	no	no	no
**8**	yes	yes	yes	yes	yes	yes	yes	yes	yes	yes	yes	yes	yes	yes	yes	yes	yes	yes	yes	yes	yes	yes	yes	yes	yes
**9**	NA	NA	NA	NA	NA	NA	NA	NA	NA	NA	NA	NA	NA	NA	NA	NA	NA	NA	NA	NA	NA	NA	NA	NA	NA
**10**	yes	yes	yes	yes	yes	yes	yes	yes	yes	yes	yes	yes	yes	yes	yes	yes	yes	yes	yes	yes	yes	yes	yes	yes	yes
**11**	NR	NR	NR	NR	NR	NR	NR	NR	NR	NR	NR	NR	NR	NR	NR	NR	NR	NR	NR	NR	NR	NR	NR	NR	NR
**12**	yes	yes	no	yes	no	no	yes	yes	yes	no	yes	no	yes	no	no	yes	yes	yes	yes	no	yes	no	no	no	yes
**Quality rating**	Fair	Fair	Fair	Fair	Fair	Fair	Fair	Fair	Fair	Fair	Good	Good	Good	Good	Good	Good	Good	Good	Fair	Good	Good	Good	Fair	Good	Good

B

	Huang, Y. et al., 2021	Ji, L. et al., 2021	Nakai et al., 2021	Sawicka-Smiarowska et al. 2021	Stø et al., 2021	Sun et al., 2021	Tabata et al., 2021	Tian et al. 2021	Wang et al. 2021	Xu et al., 2021	Chen et al. 2022	Chiu et al., 2022	Choroszy et al. 2022	Li, W et al. 2022	Liu, C. et al., 2022	Liu, L. et al. 2022	Liu, X. et al. 2022	Luo, J. et al. 2022	Qian et al., 2022	Tang et al., 2022	Tian et al., 2022	Yu et al. 2022	Zhao et al., 2022	Zheng et al., 2022	Zhong et al. 2022
**1**	yes	yes	yes	yes	yes	yes	yes	yes	yes	yes	yes	yes	yes	yes	yes	yes	yes	yes	yes	yes	yes	yes	yes	yes	yes
**2**	yes	yes	yes	yes	yes	yes	yes	yes	yes	yes	yes	yes	yes	yes	yes	yes	yes	yes	yes	yes	yes	yes	yes	yes	yes
**3**	no	no	no	no	no	no	no	no	no	no	no	no	no	no	no	no	no	no	no	no	no	no	no	no	no
**4**	yes	yes	yes	yes	yes	yes	yes	yes	yes	yes	yes	yes	yes	yes	yes	yes	yes	yes	yes	yes	yes	yes	yes	yes	yes
**5**	yes	yes	yes	yes	yes	yes	yes	yes	yes	yes	yes	yes	yes	yes	yes	yes	yes	yes	yes	yes	yes	yes	yes	yes	yes
**6**	yes	yes	yes	yes	yes	yes	yes	yes	yes	yes	yes	yes	yes	yes	yes	yes	yes	yes	yes	yes	yes	yes	yes	yes	yes
**7**	no	no	no	no	no	no	no	no	no	no	no	no	no	no	no	no	no	no	no	no	no	no	no	no	no
**8**	yes	yes	yes	yes	yes	yes	yes	yes	yes	yes	yes	yes	yes	yes	yes	yes	yes	yes	yes	yes	yes	yes	yes	yes	yes
**9**	NA	NA	NA	NA	NA	NA	NA	NA	NA	NA	NA	NA	NA	NA	NA	NA	NA	NA	NA	NA	NA	NA	NA	NA	NA
**10**	yes	yes	yes	yes	yes	yes	yes	yes	yes	yes	yes	yes	yes	yes	yes	yes	yes	yes	yes	yes	yes	yes	yes	yes	yes
**11**	NR	NR	NR	NR	NR	NR	NR	NR	NR	NR	NR	NR	NR	NR	NR	NR	NR	NR	NR	NR	yes	NR	NR	NR	NR
**12**	no	yes	yes	yes	no	no	no	yes	no	yes	no	no	no	yes	yes	no	yes	yes	no	yes	yes	no	no	no	yes
**Quality rating**	Fair	Fair	Good	Fair	Fair	Good	Fair	Good	Fair	Good	Fair	Fair	Fair	Good	Fair	Fair	Good	Fair	Fair	Fair	Good	Good	Good	Fair	Fair

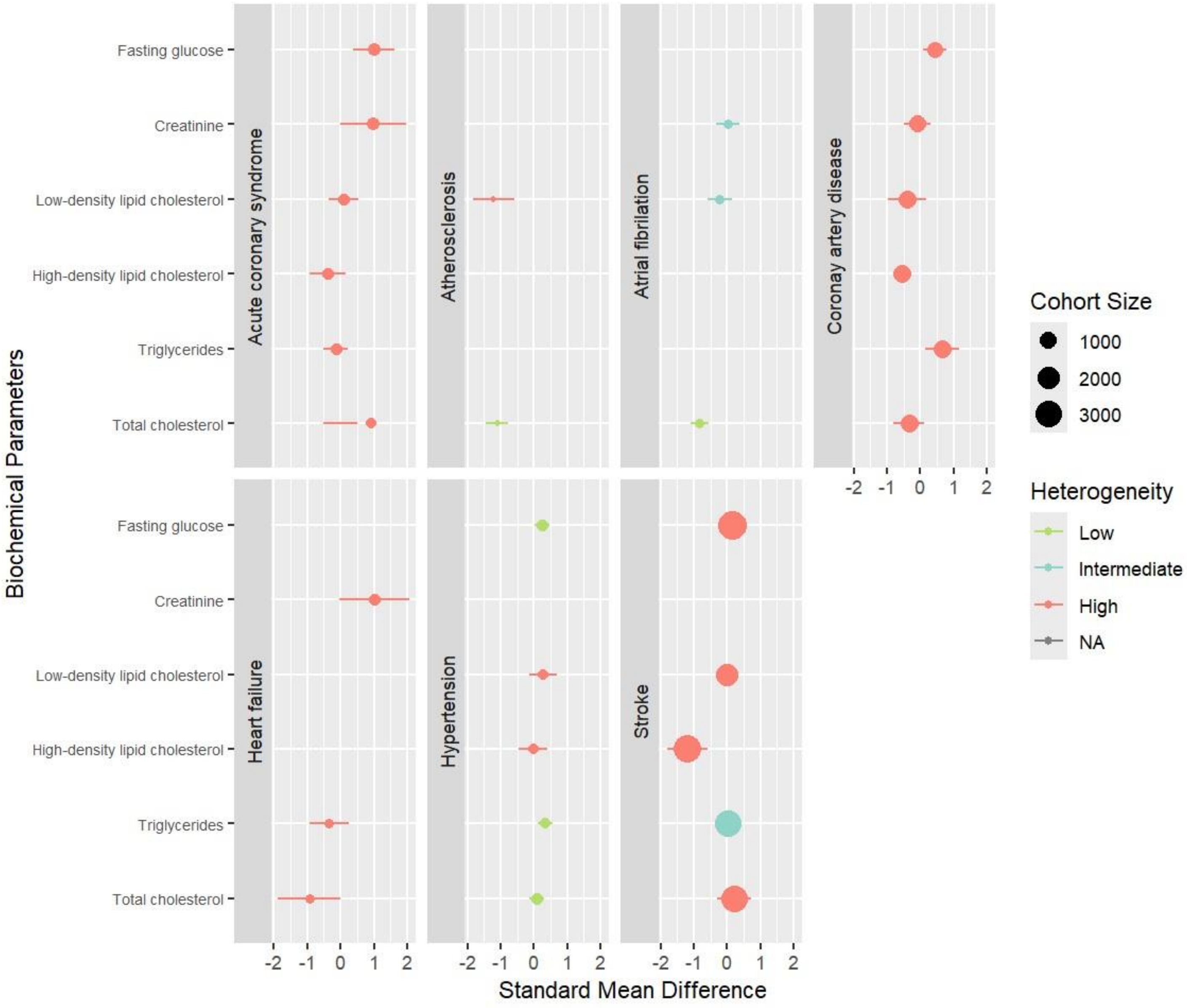

Figure S1. Biochemical data across different types of cardiovascular disease (CVD).

Forest plot analysis of standard mean differences (SMD) in biochemical data between patients with CVD and healthy controls (HC). The size of each circle corresponds to the cohort size, reflecting study participant numbers. Horizontal lines represent 95% confidence intervals for individual study results. The heterogeneity level is indicated by colour coding: I^2 values of less than 25% (low, light green), 25-50% (intermediate, light blue), and over 50% (high, light red).
